# Targeting stress induction of GRP78 by cardiac glycoside oleandrin dually suppresses cancer and COVID-19

**DOI:** 10.1186/s13578-024-01297-3

**Published:** 2024-09-06

**Authors:** Dat P. Ha, Woo-Jin Shin, Ze Liu, Michael E. Doche, Roy Lau, Nektaria Maria Leli, Crystal S. Conn, Mariangela Russo, Annalisa Lorenzato, Constantinos Koumenis, Min Yu, Shannon M. Mumenthaler, Amy S. Lee

**Affiliations:** 1 Department of Biochemistry and Molecular Medicine, Keck School of Medicine, University of Southern California, Los Angeles, CA 90033 USA; 2 Norris Comprehensive Cancer Center, Keck School of Medicine, University of Southern California, Los Angeles, CA 90033 USA; 3 Florida Research and Innovation Center, Cleveland Clinic, Port St. Lucie, FL 34987 USA; 4 Department of Cancer Biology, Infection Biology Program, and Global Center for Pathogen and Human Health Research, Lerner Research Institute, Cleveland Clinic, Cleveland, OH 44106 USA; 5 Ellison Institute of Technology, Los Angeles, CA 90064 USA; 6 Department of Radiation Oncology, The Perelman School of Medicine, University of Pennsylvania, Philadelphia, PA 19104 USA; 7 Dipartimento di Oncologia, Molecular Biotechnology Center, Università di Torino, Turin, Italy; 8 Department of Stem Cell Biology and Regenerative Medicine, Keck School of Medicine, University of Southern California, Los Angeles, CA 90033 USA; 9 Department of Medicine, Keck School of Medicine, University of Southern California, Los Angeles, CA 90033 USA

**Keywords:** GRP78, ER stress, Cardiac glycosides, Oleandrin, SARS-CoV-2, Colon cancer, Breast cancer, Anti-cancer therapy, Anti-viral therapy

## Abstract

**Background:**

Despite recent therapeutic advances, combating cancer resistance remains a formidable challenge. The 78-kilodalton glucose-regulated protein (GRP78), a key stress-inducible endoplasmic reticulum (ER) chaperone, plays a crucial role in both cancer cell survival and stress adaptation. GRP78 is also upregulated during SARS-CoV-2 infection and acts as a critical host factor. Recently, we discovered cardiac glycosides (CGs) as novel suppressors of GRP78 stress induction through a high-throughput screen of clinically relevant compound libraries. This study aims to test the possibility that agents capable of blocking stress induction of GRP78 could dually suppress cancer and COVID-19.

**Results:**

Here we report that oleandrin (OLN), is the most potent among the CGs in inhibiting acute stress induction of total GRP78, which also results in reduced cell surface and nuclear forms of GRP78 in stressed cells. The inhibition of stress induction of GRP78 is at the post-transcriptional level, independent of protein degradation and autophagy and may involve translational control as OLN blocks stress-induced loading of ribosomes onto *GRP78* mRNAs. Moreover, the human Na^+^/K^+^-ATPase α3 isoform is critical for OLN suppression of GRP78 stress induction. OLN, in nanomolar range, enhances apoptosis, sensitizes colorectal cancer cells to chemotherapeutic agents, and reduces the viability of patient-derived colon cancer organoids. Likewise, OLN, suppresses GRP78 expression and impedes tumor growth in an orthotopic breast cancer xenograft model. Furthermore, OLN blocks infection by SARS-CoV-2 and its variants and enhances existing anti-viral therapies. Notably, GRP78 overexpression mitigates OLN-mediated cancer cell apoptotic onset and suppression of virus release.

**Conclusion:**

Our findings validate GRP78 as a target of OLN anti-cancer and anti-viral activities. These proof-of-principle studies support further investigation of OLN as a readily accessible compound to dually combat cancer and COVID-19.

**Supplementary Information:**

The online version contains supplementary material available at 10.1186/s13578-024-01297-3.

## Introduction

Despite recent advances in cancer therapy based on groundbreaking discoveries in pharmaceutical and immunologic approaches, the development of resistance and metastatic growth remains a major challenge and contributes to the fatality of the disease. Thus, it is critical to identify host factors which enable cancer cells to adapt to stresses associated with tumorigenesis and therapeutic treatment to gain a survival advantage.

Cancer cells are often in a state of sustained stress due to rapid proliferation and altered metabolism, coupled with hypoxia and nutrient deprivation due to insufficient vascularization, and they utilize multiple stress responses to cope with these adverse conditions [1–3]. The 78-kilodalton glucose-regulated protein (GRP78), also referred to as BiP and encoded by the *HSPA5* gene, is a key endoplasmic reticulum (ER) chaperone and a master regulator of the unfolded protein response (UPR), which is an intracellular quality control system that senses harmful malfolded proteins accumulating in the ER and triggers a cascade of adaptive pathways [4–7]. For survival, cancer cells usurp the protective aspects of the UPR, of which a key feature is induction of GRP78, a multifunctional protein with potent anti-apoptotic functions, thereby enabling them to overcome innate immunity and therapy-induced cell death [8]. As such, GRP78 is commonly upregulated in a wide range of cancers and is critical for cancer cell survival, metastasis, and resistance to therapy [8–12]. The pro-survival effects of GRP78 are observed in proliferating and dormant cancer cells, tumor initiating cells and in tumor-associated endothelial cells that supply nutrients and oxygen to the tumor cells. They involve not only the ER form of GRP78, but also the stress-induced cytosolic isoform, the secreted form, and the cell surface form of GRP78 (csGRP78) [13–16]. The latter is particularly important since the translocation of GRP78 from the ER to the cell surface is actively promoted by stress, and on the cell surface, csGRP78 assumes novel functions beyond the ER and regulates multiple oncogenic pathways, including the PI3K/AKT pathway, which is a major driver of tumor proliferation, resistance and metastasis [8, 17–23]. Recently, we discovered that GRP78 can translocate to the nucleus in stressed and malignant cells to assume a new role as a transcriptional regulator, leading to reprogramming of the cancer cell’s transcriptional activities to adopt a migratory and invasive phenotype [24]. Thus, agents capable of suppressing the stress induction of GRP78 are attractive candidates for mitigating cancer cell survival, resistance and metastasis.

In addition to its importance in cancer, GRP78 acts as a critical host factor for coronaviruses including SARS-CoV-2, with the latter being the causative agent of the COVID-19 global pandemic [25, 26]. GRP78 is upregulated both intracellularly and at the cell surface during SARS-CoV-2 infection, which elicits ER stress as the virus commandeers the host cell’s machinery for viral protein production and assembly [27, 28]. In a positive feedback cycle, ER stress promotes the translocation of GRP78 from the ER to the cell surface, where it interacts with the CoV Spike protein and host receptor protein angiotensin-converting enzyme 2 (ACE2), facilitating viral cell surface attachment and entry [26]. Importantly, GRP78 knockdown or inhibition of its activity suppressed SARS-CoV-2 Spike production, viral entry, replication and infectivity in both cell cultures and mice following viral infection, associating with decreased viral load, ameliorated lung pathology and prolonged survival [26, 28–30]. While the rapid development and deployment of vaccines have significantly mitigated the severity and mortality associated with COVID-19, challenges in maintaining the cold-chain for vaccine storage and distribution to developing countries and emergence of novel viral variants highlight the need for improving vaccination strategies and treatment modalities [31, 32]. Considering this, therapeutic agents that can suppress stress induction of GRP78 will deprive the virus of an essential chaperone for their entry and viral protein production, with the added advantage that GRP78 is more stable and less susceptible to mutation than the rapidly evolving virus itself.

These raise the interesting possibility that a therapeutic agent capable of blocking the stress induction of GRP78 could dually suppress cancer and COVID-19. Towards that goal and fast-tracking to the clinic, we recently reported a high-throughput screen of clinically relevant compound libraries yielding a surprising result that many of the top hits belonged to a class of compounds referred to as cardiac glycosides (CGs) and they could inhibit ER-stress induction of GRP78 in a variety of human cancers [33]. While CGs such as digoxin and lanatoside C (LanC) are FDA-approved to treat heart failures, they were subsequently found to also exhibit anti-cancer activities both in cell cultures and xenograft models, with diverse mechanisms implicated for their anti-tumor activities [34–38]. For instance, digoxin has been reported to suppress the expression of HIF-1α, which is an important protein for both cancer and SARS-CoV-2 infection [36, 39, 40]. In this study, we focused on oleandrin (OLN), a unique lipid-soluble CG derived from *Nerium oleander.* Among the CGs, OLN is the most potent exhibiting a 100-fold higher affinity for the α3 versus α1 isoform of the Na^+^/K^+^-ATPase and higher tolerability [41], and PBI-05204 which contains OLN as its active ingredient, exhibited a safe profile in human Phase I/II clinical trials in cancer patients [42, 43]. Furthermore, OLN has been reported to exhibit antiviral activity specifically against enveloped viruses including HIV, HTLV-1, and SARS-CoV-2 [44, 45]. However, the mechanisms may be context-dependent and remain to be determined.

Here, we established that OLN in nanomolar range can dually suppress cancer and SARS-CoV-2 and investigated into its mechanisms. OLN can suppress the induction of GRP78 by a variety of ER stress conditions in cancer cell cultures, including those that have developed drug resistance, leading to the onset of apoptosis. In human lung epithelial cells infected with SARS-CoV-2 or its variants including Omicron lineage, OLN suppresses GRP78 induction and viral production without affecting cell viability and enhanced the activity of antiviral agents Remdesivir and Nirmatrelvir in combination therapy. These proof-of-principle studies support further investigation of OLN and its derivatives to dually combat cancer and COVID-19.

## Results

### Oleandrin exhibits the highest potency among cardiac glycosides in blocking stress induction of GRP78 in cancer cells

To evaluate the suitability of OLN as a suppressor of GRP78 stress induction in cancer, we first compared the potency of OLN to other CGs. Human colorectal (HCT116) and breast cancer (MDA-MB-231) cell lines were selected as the experimental models, since analysis of The Cancer Genome Atlas (TCGA) database revealed significantly elevated *GRP78* mRNA expression across all subtypes of both cancer types compared to normal tissues (Figure S1A-D). Recently we reported that the CG LanC can block ER stress induction of GRP78 in the micromolar range [33]. Initial testing in HCT116 cells treated with increasing concentrations (10 to 100 nM) of OLN and LanC showed that OLN could suppress thapsigargin (Tg) induction of GRP78 at concentrations as low as 20 nM, while LanC showed no effect even at 100 nM (Figure S2A). We also noted that within the 24 h treatment period, basal GRP78 level in the non-stressed cells were not affected by either CGs, and the same results were observed for the microsatellite stable HT29 and the microsatellite unstable HCT116 colorectal cancer cell lines (Figure S2B).

**Fig. 1:**
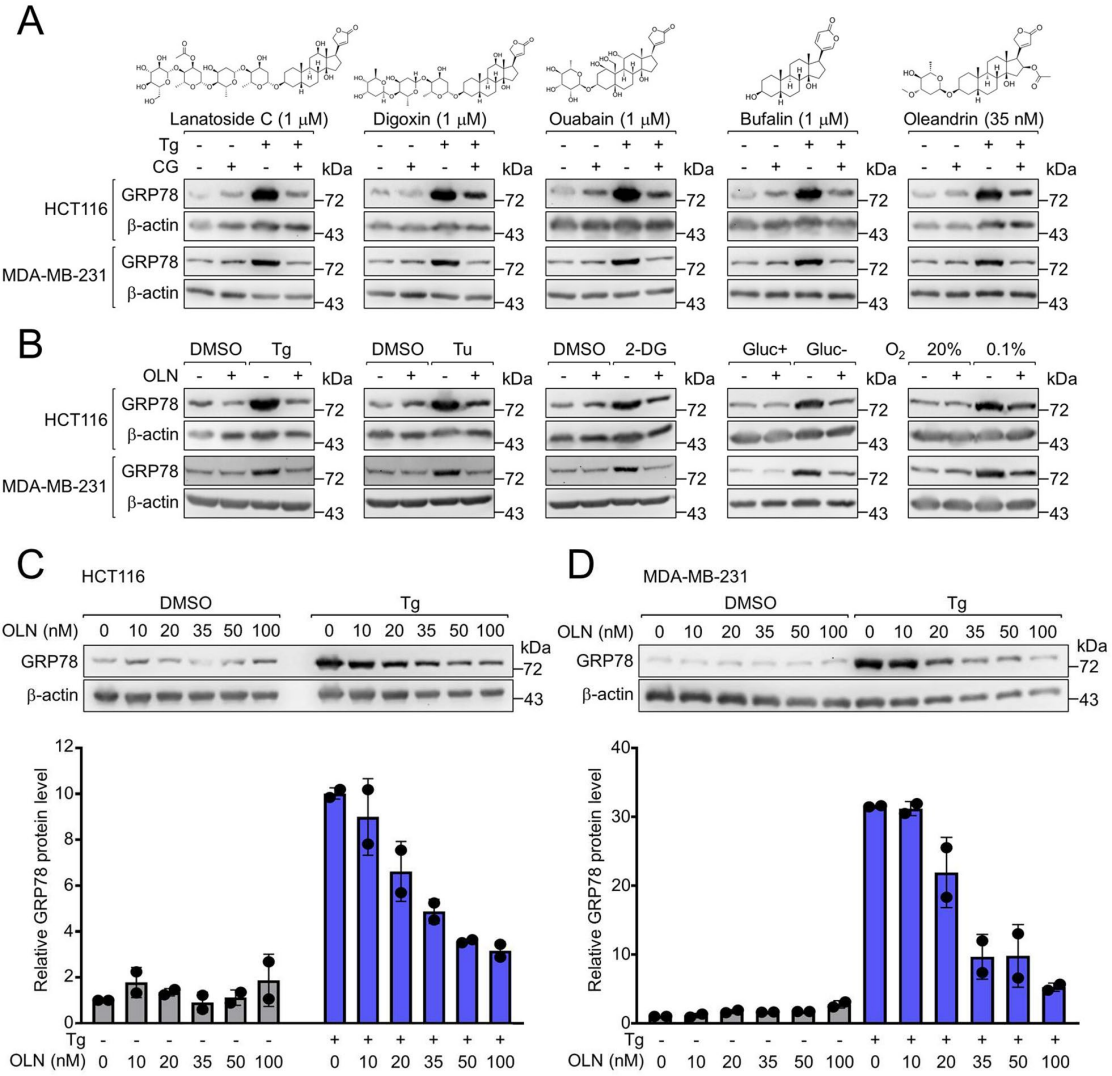
Oleandrin inhibits induction of GRP78 by multiple stress inducers in human cancer cell lines. **A** Human colorectal carcinoma cell line HCT116 or triple-negative breast cancer cell line MDA-MB-231 were treated with 1 μM of lanatoside C, digoxin, ouabain, bufalin or 35 nM of oleandrin alone or in combination with the ER stress inducer Tg (300 nM) for 24 h. Whole cell lysates (WCLs) were subjected to Western blot analysis for GRP78 protein level with β-actin serving as loading control. **B** Same as in (**A**) except the cells were treated with 35 nM of oleandrin (OLN) alone or in combination with the followings: Tg (300 nM), Tu (1.78 mM), 2-DG (10 mM), DMEM containing 4.5 mg/ml glucose (Glu +), glucose-free DMEM (Glu-), 20% O_2_, or 0.1% O_2_. **C** HCT116 cells were treated with OLN (from 10 to 100 nM) alone or in combination with Tg (300 nM) for 24 h. WCLs were subjected to Western blot analysis for GRP78 protein level with β-actin serving as loading control. Quantitation of the relative levels of GRP78 normalized to β-actin are shown in the graphs below. **D** Same as in (**C**) except MDA-MB-231 cells were used. Data are presented as mean ± S.D

Next, in addition to LanC, we tested the potency of OLN in comparison to three additional CGs (digoxin, ouabain and bufalin) in both HCT116 and MDA-MB-231 cells. We observed that in both cancer types, OLN at 35 nM was as effective as the other CGs at 1 μM in suppressing Tg stress induction of GRP78 (Fig. 1A). In addition to Tg, OLN also effectively blocked GRP78 induction by other ER stress inducers including tunicamycin (Tu) and 2-Deoxy-d-glucose (2-DG), as well as glucose starvation and hypoxia commonly observed during tumorigenesis (Fig. 1B). We further determined that for HCT116, HT29 and MDA-MB-231 cells, the EC_50_ for OLN blockage of Tg induction of GRP78 was approximately 25 to 35 nM for the three cell lines (Fig. 1C, D and S2B). Notably, the OLN suppressive effect is most prominent with GRP78, which is highly inducible by Tg, followed by a mild effect on GRP94, which is moderately inducible by Tg, while OLN showed no effect on the other ER chaperones (calnexin and PDI) and the cytosolic chaperone HSP70 that are not inducible by Tg within the 24 h treatment period (Figure S2C and D). Collectively, these results reveal that OLN is highly effective in inhibiting the acute induction of GRP78 by stress.

### Suppression of stress induction of total GRP78 by oleandrin resulted in reduced GRP78 expression at the cell surface and nucleus

**Fig. 2:**
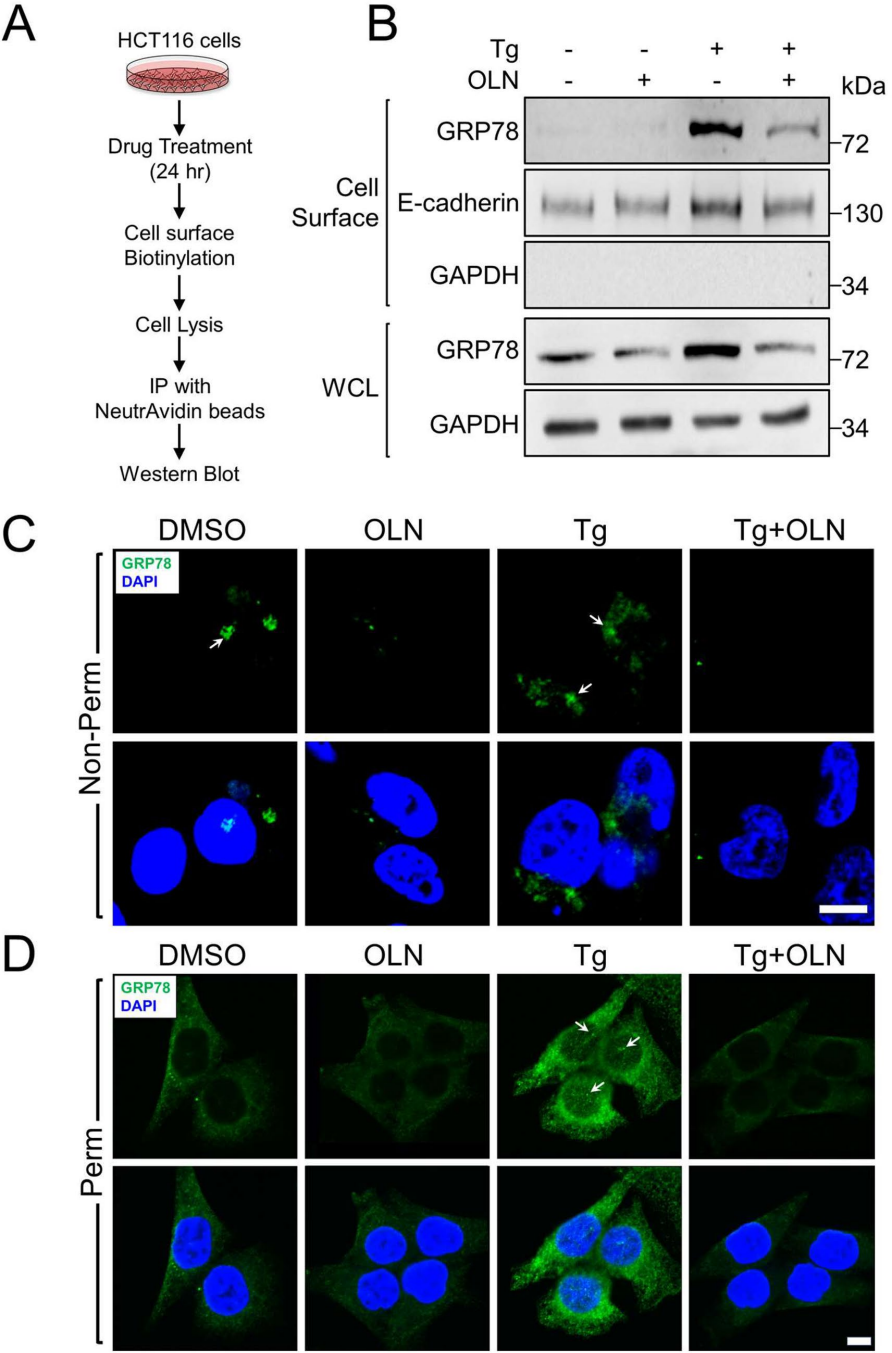
Oleandrin treatment suppresses stress induced total, cell surface and nuclear GRP78 expression. **A** Experimental scheme to purify and detect GRP78 on the cell surface. **B** HCT116 cells were treated with OLN (35 nM) alone or in combination with Tg (300 nM) for 24 h. The cells were then subjected to the cell surface protein purification procedure outlined in (**A**). Whole cell lysate (WCL) and cell surface fractions were subjected to Western blot analysis for GRP78 protein level with E-cadherin and GAPDH serving as loading controls for cell surface and total proteins respectively. **C** Same as in (**B**) except the cells were subjected to confocal immunofluorescence staining for GRP78 without permeabilization. The nuclei were stained by DAPI in blue. The white arrows indicate GRP78 on the cell surface. **D** Same as in (**C**) except the cells were permeabilized and stained for GRP78. The white arrows indicate GRP78 in the nucleus. In both (**C**) and (**D**), the scale bars represent 20 μm

In addition to its canonical localization in the ER, under stress, GRP78 has been reported to translocate to various cellular compartments, including the cell surface and nucleus, where it exerts multiple new functions [15, 24]. To test the effect of OLN on GRP78 expression on the cell surface under stress, HCT116 cells were treated with 35 nM of OLN, either alone or in combination with Tg. Cell surface proteins were labeled by biotinylation and isolated through immunoprecipitation and the level of cell surface GRP78 (csGRP78) was determined by Western blot (Fig. 2A). As expected, Tg greatly increased the level of both total and csGRP78, and both forms of GRP78 were reduced when the cells were treated with Tg in combination with OLN (Fig. 2B). These biochemical findings were further corroborated through immunofluorescence staining of HCT116 cells under non-permeabilized conditions to detect csGRP78 (Fig. 2C). To assess the impact of OLN on stress induced nuclear GRP78 (nuGRP78) expression, HCT116 cells were treated with 35 nM of OLN, either alone or in combination with Tg, followed by immunofluorescence staining with permeabilization to detect both total and nuGRP78 expression. The confocal microscopy images showed that Tg treatment led to a substantial increase in both total and nuGRP78 expression (Fig. 2D). The combined treatment of Tg and OLN resulted in a marked suppression of both total and nuGRP78 expression (Fig. 2D). Thus, OLN, through inhibition of stress induction of total GRP78, also led to reduced expression of the cell surface and nuclear forms of GRP78 in stressed cells.

### Oleandrin suppresses acute stress induction of GRP78 via post-transcriptional mechanism independent of protein degradation and autophagy

**Fig. 3:**
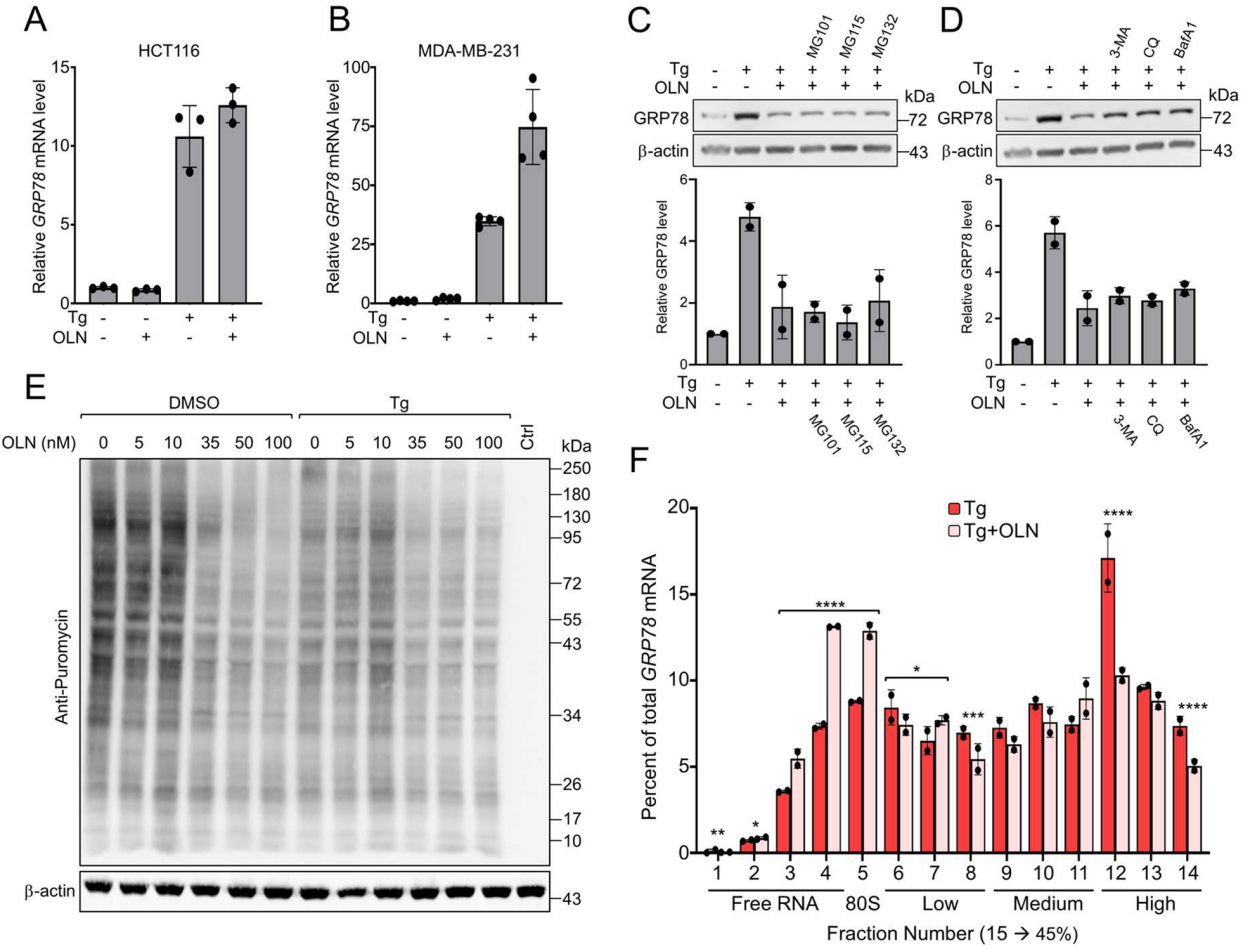
Oleandrin suppresses GRP78 stress induction at the post-transcriptional level, independent of protein degradation and autophagy. **A** HCT116 cells were treated with OLN (35 nM) alone or in combination with Tg (300 nM) for 24 h. *GRP78* mRNA level was determined by RT-qPCR and its relative level was graphed with *β-actin* mRNA serving as loading control. **B** Same as in (**A**) except MDA-MB-231 cells were used. **C** HCT116 cells were treated as in (**A**) and three different proteasome inhibitors MG101 (10 μM), MG115 (10 μM) or MG132 (10 μM) for 24 h. Western blot analysis was performed for GRP78 protein level with β-actin serving as loading control. Quantitation of the relative levels of GRP78 normalized to β-actin are shown in the graphs below. **D** Same as in (**C**) except the cells were treated in combination with three different autophagy inhibitors 3-MA (10 mM), Chloroquine (CQ, 20 μM), or Bafilomycin A1 (BafA1, 100 nM). **E** HEK293T cells were treated with the indicated concentrations of OLN and Tg, alone or in combination for 8 h followed by 30 min incubation with puromycin (4 μg/ml). Western blot for puromycin labeled proteins is shown with β-actin serving as loading control. **F** Cells were treated with OLN (30 nM) alone or in combination with Tg (300 nM) for 6 h. Cell lysates were sedimented on sucrose gradient followed by fractionation. RNA was extracted from each fraction and subject to RT-qPCR analysis of *GRP78* mRNA. The value on the y-axis corresponds to the percentage of total *GRP78* mRNA across fractions. Indicated below the fractions are the free ribosomal subunits which consist of the lower fractions (< 5), the 80S monosome, the low polysome fractions 6 to 8, the medium fractions 9 to 11, and the high polysome fractions 12 to 14 (n = 6, 6 technical replicates from two biological experiments). Data are presented as mean ± S.D. *p ≤ 0.05, **p ≤ 0.01, ***p ≤ 0.001, ****p ≤ 0.0001 (Student’s *t* test)

In deciphering the mechanism(s) through which OLN suppresses stress induction of GRP78, we observed that OLN treatment either had no effect or even increased *GRP78* mRNA levels in Tg-treated HCT116 and MDA-MB-231 cells respectively (Fig. 3A and B). Co-treatment of HCT116 cells with proteasome inhibitors (MG101, MG115, and MG132) or autophagy inhibitors (3-MA, Chloroquine, and Bafilomycin A1) in combination with Tg and OLN also did not restore GRP78 protein levels induced by Tg (Fig. 3C and D). Thus, the OLN effect is unlikely due to transcriptional block or protein degradation. To test whether translational regulation was involved, we performed a puromycin pulse experiment using HEK293T cells as a model system and observed that OLN at 35 nM or higher mildly suppressed the production of newly synthesized proteins in cells that were either non-treated or treated with Tg for 8 h (Fig. 3E), consistent with a previous report that CGs can inhibit general protein synthesis [46]. To examine the effect of OLN on GRP78 in a human cancer cell line, we performed polysome profiling of *GRP78* mRNA in HCT116 cells treated with Tg alone or in combination with OLN. We observed significant reduction in the loading of ribosomes onto *GRP78* mRNA, coupled with an increase of free *GRP78* mRNA and in association with the 80S ribosomal subunit (Fig. 3F). Collectively these results suggest that among the diverse effects of OLN on cellular homeostassis, OLN suppressed acute stress induction of GRP78 at the post-transcriptional level independent of protein degradation or autophagy and may involve translational attenuation.

### Oleandrin suppression of acute stress induction of GRP78 requires the human Na^+^/K^+^-ATPase α3 isoform.

**Fig. 4:**
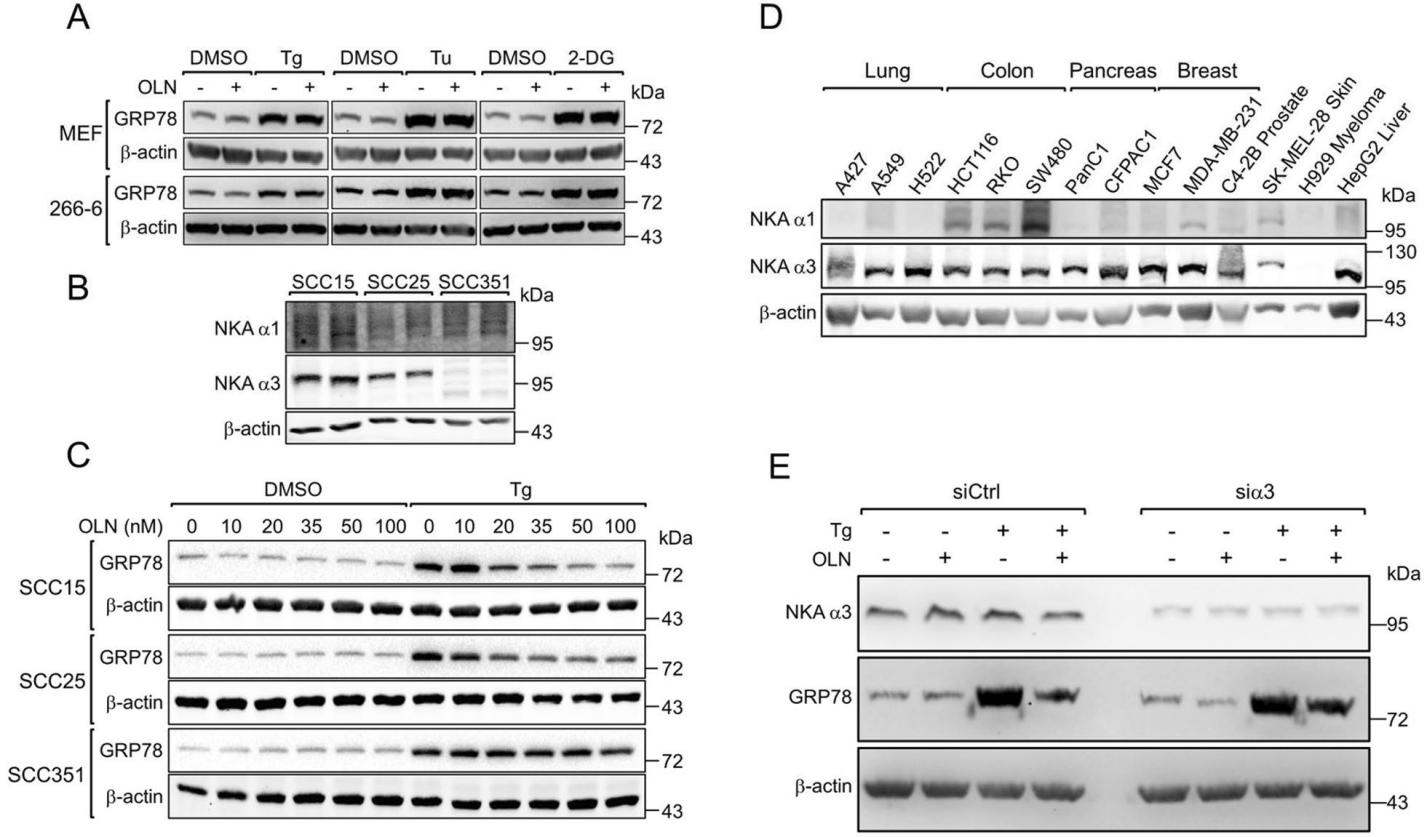
Oleandrin suppresses stress induction of GRP78 requires the human Na^+^/K^+^-ATPase α3 isoform. **A** Mouse embryonic fibroblasts (MEFs) or mouse acinar pancreatic cancer cells 266–6 were treated with 35 nM of OLN alone or in combination with different ER stress inducers Tg (300 nM), Tu (1.78 mM), or 2-DG (10 mM) for 24 h. Whole cell lysates (WCLs) were subjected to Western blot analysis for GRP78 protein level with β-actin serving as loading control. **B** Relative protein expression levels of the NKA α1 and α3 isoforms in a panel of 3 head and neck cancer cell lines (SCC15, SCC25, and SCC351). Whole cell lysates were analyzed by Western blot with β-actin serving as loading control. **C** SCC15, SCC25, and SCC351 cells were treated with OLN (from 10 to 100 nM) alone or in combination with Tg (300 nM) for 24 h. WCLs were subjected to Western blot analysis for GRP78 protein level with β-actin serving as loading control. **D** Relative protein expression levels of the Na^+^/K^+^-ATPase α1 (NKA α1) and α3 (NKA α3) isoforms in a panel of 14 different cancer cell lines. Whole cell lysates were analyzed by Western blot with β-actin serving as loading control. **E** HCT116 cells were transfected with control siRNA (siCtrl) or siRNA targeting the NKA α3 isoform (siα3) for 24 h. The cells were then treated with OLN (35 nM) alone or in combination with Tg (300 nM) for an additional 24 h. WCLs were subjected to Western blot analysis for GRP78 and NKA α3 protein levels with β-actin serving as loading control

Interestingly, it has been reported that CGs can inhibit general protein synthesis via inhibition of the Na^+^/K^+^-ATPase pump [46]. Furthermore, the expression and cellular location of the α3 subunit of the Na^+^/K^+^-ATPase pump influences the anti-proliferative activity of OLN [41]. Taking advantage of mutations in the murine α3 gene that render mouse cells insensitive to CGs action [47], we showed that all five CGs including OLN failed to suppress Tg induction of GRP78 in two different mouse cell lines (Figure S3A). In both murine cell lines, OLN treatment did not affect stress induction of GRP78 by different ER stress inducers (Fig. 4A). Furthermore, we utilized a human head and neck cancer cell line SCC351, which expresses a much lower level of Na^+^/K^+^-ATPase α3 isoform compared to the SCC15 and SCC25 cell lines, with similar levels of the α1 isoform in all 3 cell lines (Fig. 4B). While Tg increased GRP78 levels in all 3 cell lines, the suppressive effect of OLN was only evident in SCC15 and SCC25, but not in SCC351 (Fig. 4C). In contrast to the α1 isoform, we observed widespread expression of the α3 isoform across a wide range of cancer types (Fig. 4D). To directly test the importance of the α3 isoform, we knocked down its expression via specific siRNA in HCT116 cells and observed that the suppressive effect of OLN on GRP78 induction was attenuated compared to cells treated with control siRNA (Fig. 4E). To determine if this rescue effect occurs at the transcriptional level, we isolated RNA from the same experiment and performed RT-qPCR to measure *GRP78* mRNA levels. We found that α3 knockdown did not significantly affect *GRP78* mRNA levels under OLN and Tg treatment, alone or in combination, compared to control siRNA, suggesting that OLN suppresses GRP78 induction via the α3 isoform post-transcriptionally (Figure S3B). Taken together, these results indicate that the integrity of the Na^+^/K^+^-ATPase α3 isoform, abundantly expressed in a wide range of cancer types, is critical for the OLN effect on GRP78 stress induction.

### Oleandrin sensitizes colorectal cancer cells to chemotherapeutic agents and reduces the viability of patient-derived colon cancer organoids

**Fig. 5:**
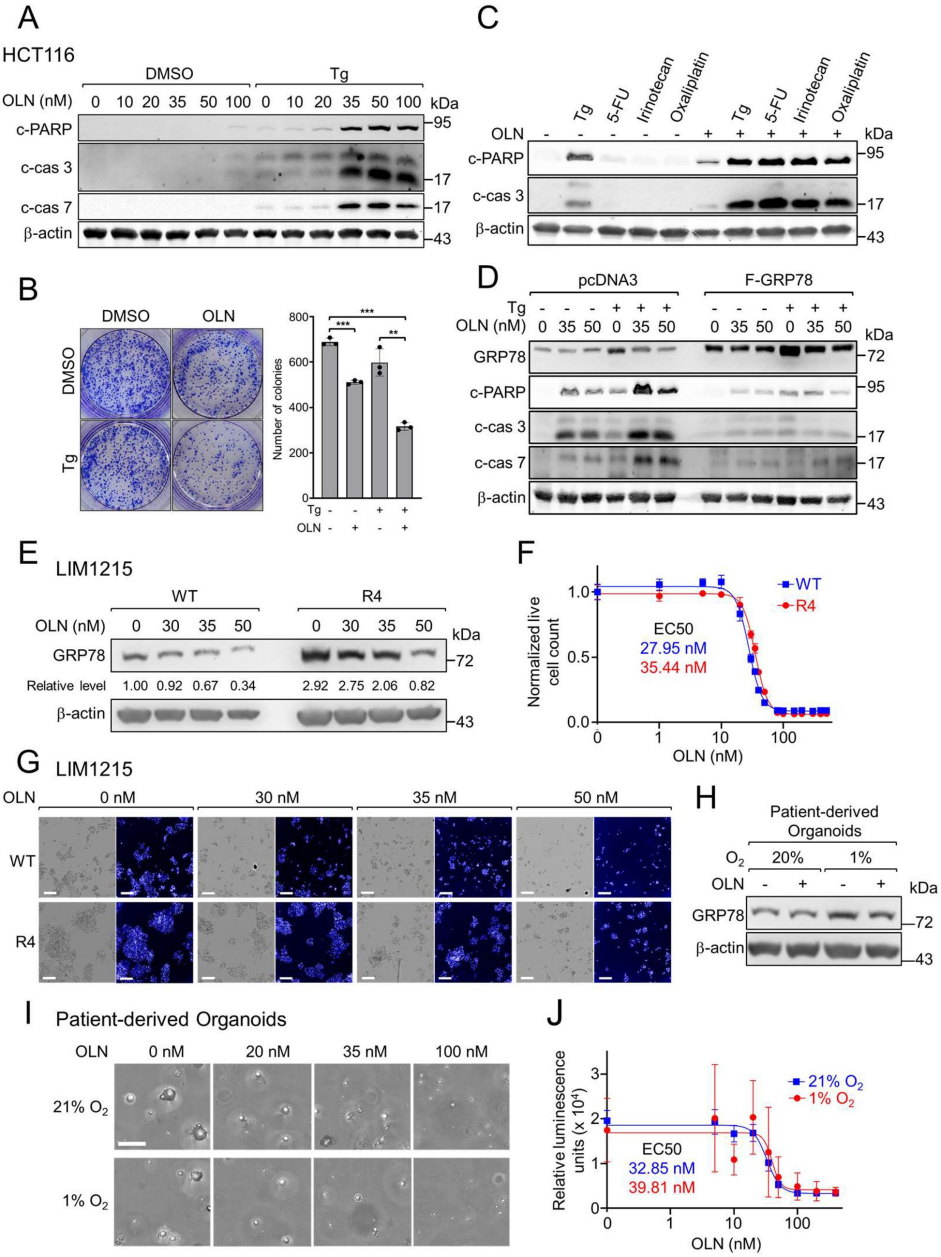
Oleandrin sensitizes colorectal cancer cells to chemotherapeutic agents and reduces viability of patient-derived organoids. **A** HCT116 cells were treated with OLN and Tg as indicated for 24 h. Western blot was performed for the cleaved forms of PARP (c-PARP), caspase-3 (c-cas 3), or caspase-7 (c-cas 7) and β-actin. **B** HCT116 cells were treated with OLN (35 nM) and Tg (300 nM) as indicated for 24 h. Colonies formed after 2 weeks were stained by Coomassie blue and quantitated. Data are presented as mean ± S.D. **p ≤ 0.01, ***p ≤ 0.001 **C** HCT116 cells were treated with Tg (300 nM), 5-FU (5 μM), Irinotecan (5 μM), Oxaliplatin (1 μM), and OLN (35 nM) as indicated for 24 h. Western blot was performed for the indicated proteins. **D** HCT116 cells were transfected with the indicated vectors for 24 h, then treated with OLN and Tg (300 nM) as indicated for an additional 24 h. Western blot was performed for the indicated proteins. **E** LIM1215 Cetuximab-sensitive (WT) and resistant (R4) cells were treated with OLN as indicated for 72 h and probed for GRP78 and β-actin with GRP78 level normalized to β-actin shown below. (**F**) Cell viability measured by live cell counts using image analysis segmentation with values normalized to 0 nM OLN. Means ± S.D. with non-linear regression best-fit curves were plotted with respective EC_50_ values noted (n = 6 for 0 nM and n = 3 for all other concentrations). **G** Representative images of bright field and Hoechst 33342-stained cells treated with OLN for 3 days (Scale bar, 200 μm). (**H**) Colon cancer patient-derived organoids (PDOs) were treated as indicated and probed for GRP78 and β-actin levels. (**I**) Maximum projection images of the PDOs treated as indicated (Scale bar, 200 μm). (**J**) PDOs were treated as indicated and their viability was measured by CellTiter Glo 3D relative luminescence units and quantified as in (**F**) (n = 5–6 technical replicates)

Next, we assessed OLN effects on apoptosis and cell survival under stress in colon cancer. In HCT116 cells, in contrast to non-stressed cells, co-treatment of OLN with Tg led to substantial increases in cleaved PARP, caspase-3, and caspase-7 in a dose-dependent manner, indicating robust apoptosis induction (Fig. 5A). Furthermore, these results were supported by colony formation assays where combined treatment of OLN and Tg led to a statistically significant reduction of viable colonies, compared to controls (Fig. 5B). Given that GRP78 is a master regulator of the UPR, which can trigger apoptosis upon prolonged stress, we investigated whether OLN treatment activates the UPR and triggers apoptosis. In examining the effect of OLN on the UPR pathways (PERK, IRE1, and ATF6) following Tg treatment, we observed that while OLN had no effect on Tg activation of the IRE1 and ATF6 pathways, as evidenced by unaltered *XBP-1* splicing and ATF6 cleavage, it increased eIF2α phosphorylation, a marker for PERK activation (Figure S4). However, Tg induction of ATF4 and CHOP, which are downstream effectors of PERK and with CHOP being a mediator for UPR-induced apoptosis, was suppressed by OLN treatment (Figure S4). These results suggest that OLN-induced apoptosis in Tg treated cells likely does not involve the UPR.

Combination treatment of OLN with the chemotherapeutic agent 5-FU, Irinotecan, or Oxaliplatin greatly increased the levels of apoptotic markers, compared to drug treatment alone, indicating that OLN sensitized the colon cancer cells to these chemotherapeutic agents (Fig. 5C). Moreover, we showed a marked reduction in levels of the apoptotic markers (cleaved PARP, caspase-3, and caspase-7) in HCT116 cells transiently transfected with Flag-tagged GRP78 compared to those transfected with an empty vector control (pcDNA3) (Fig. 5D). These results directly demonstrated that OLN-induced cytotoxicity can be rescued by GRP78 over-expression.

We then tested OLN's efficacy on the viability of isogenic colorectal cancer cell lines, LIM1215 WT and R4, which are sensitive and resistant to Cetuximab (an anti-epidermal growth factor receptor therapy) respectively, with the resistant line R4 exhibiting higher GRP78 level (Fig. 5E). Treatment with increasing OLN concentrations resulted in dose-dependent decreases in GRP78 protein in both lines (Fig. 5E). Using high-content imaging, we quantified live cells for each condition, revealing dose-dependent reductions with EC_50_ values of 27.95 nM and 35.44 nM for the wild-type and resistant line, respectively (Fig. 5F). Nuclei staining further provided visual confirmation of the effect on live cells (Fig. 5G). Thus, OLN is effective in reducing the viability of both the drug-sensitive and resistant colorectal cancer cell lines, with higher EC_50_ for the resistant cell line expressing higher GRP78 level.

To extend these findings, we tested the effects of OLN in patient-derived colon cancer organoid (PDO) models that more closely mimic patient tumor drug response than immortalized 2D cell lines. A metastatic colon cancer PDO line (EICL-000VX) was treated with OLN (35 nM) under normoxic (21% O_2_) or hypoxic (1% O_2_) conditions, the latter commonly occurring in tumors. Western blot analysis showed that hypoxic condition induced GRP78 expression and treatment with OLN suppressed this upregulation (Fig. 5H). OLN dose–response assays were also conducted under similar conditions and treatment with increasing OLN concentrations revealed EC_50_ values of 32.85 nM and 39.81 nM for 21% and 1% O_2_ conditions, respectively (Fig. 5I, J). These results indicate OLN’s effectiveness in reducing the viability of PDO at nanomolar concentrations, even under hypoxic conditions.

### Oleandrin treatment reduces GRP78 protein level in tumor tissues, induces apoptosis, and suppresses breast cancer tumorigenesis in vivo

**Fig. 6:**
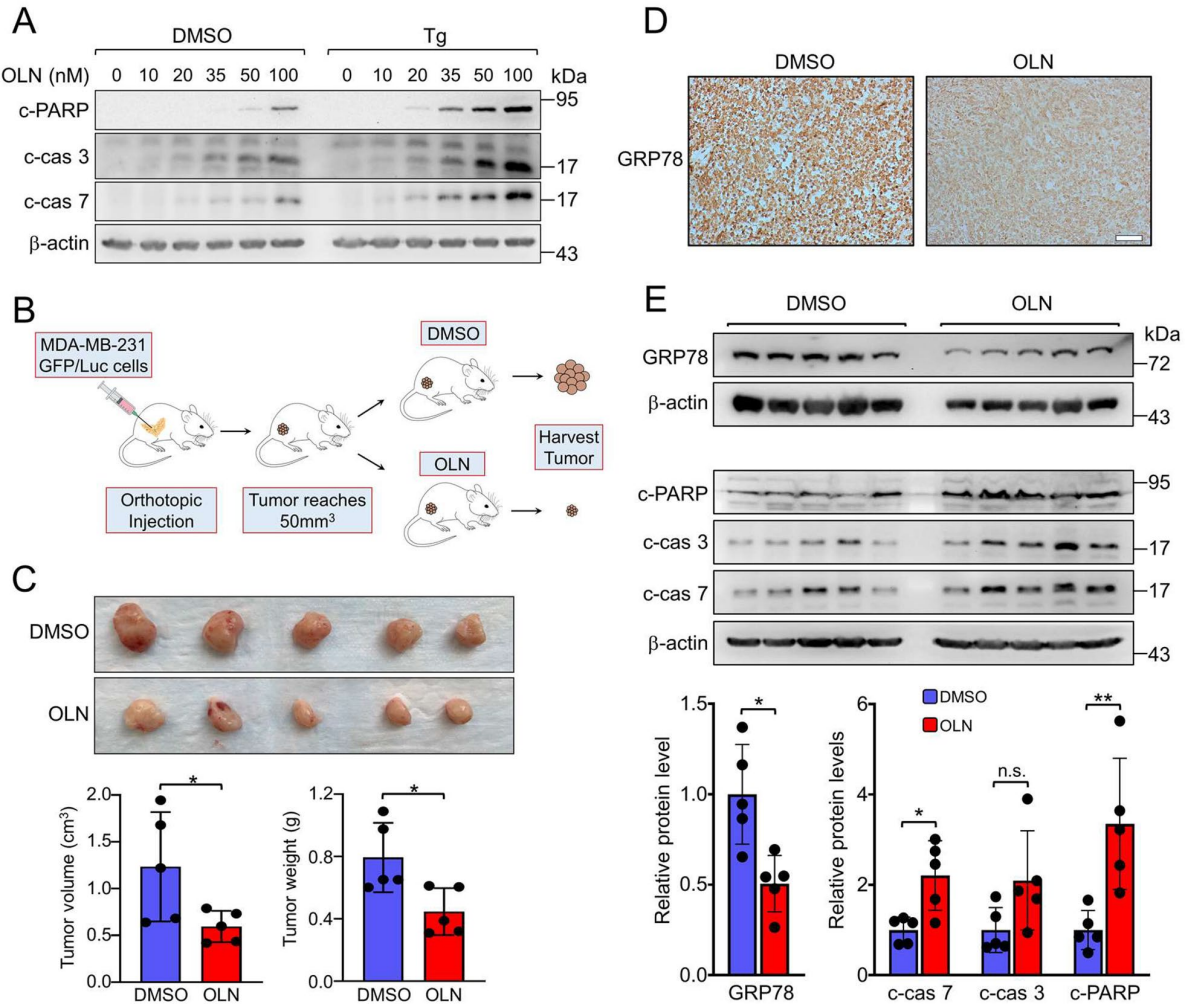
Oleandrin inhibits MDA-MB-231-GFP/Luc tumor xenograft growth and reduces GRP78 protein level in tumor tissues. **A** MDA-MB-231 cells were treated with increasing concentrations of OLN (from 10 to 100 nM) alone or in combination with Tg (300 nM) for 24 h. WCLs were subjected to Western blot analysis for the cleaved forms of PARP (c-PARP), caspase-3 (c-cas 3), or caspase-7 (c-cas 7) with β-actin serving as loading control. **B** Schematic illustration of the MDA-MB-231-GFP/Luc breast cancer cells xenograft experiment and treatment conditions. **C** Tumors formed at the end of DMSO or OLN treatment. Quantification of the tumors volume and weight is shown in the graphs below. **D** Immunohistochemical staining of the tumor xenograft tissues for GRP78 protein level (Scale bar, 100 μm). **E** Western blot analysis of tumor xenograft tissues for GRP78, c-PARP, c-cas 3, and c-cas 7 with β-actin serving as loading control. Quantitation of the relative protein levels normalized to β-actin is shown in the graphs below. Data are presented as mean ± S.D. *p ≤ 0.05, **p ≤ 0.01, n.s. denotes not significant. (Student’s *t* test)

To extend our in vitro findings to in vivo settings, we employed an orthotopic xenograft model of human breast cancer in NOD-scid gamma (NSG) mice. Initial in vitro experiments confirmed that OLN treatment led to higher levels of apoptotic markers (cleaved PARP, caspase-3, and caspase-7) in Tg-treated MDA-MB-231 cells (Fig. 6A). Following the establishment of palpable tumors (~ 50 mm^3^) in the mammary fat pads of NSG mice inoculated with MDA-MB-231-GFP/Luc cells, we initiated treatment with either vehicle (DMSO) or OLN (0.3 mg/kg) every other day for two weeks (Fig. 6B). Tumor volume and weight measurements at the endpoint revealed a significant reduction in both parameters in the OLN-treated group (Fig. 6C), in agreement with a previous study showing the same OLN dosage reduced glioma growth in vivo [48]. We further performed immunohistochemical and Western blot analyses revealing a marked decrease in tumor GRP78 protein levels upon OLN treatment (Fig. 6D, E, and S5A), coinciding with significantly elevated levels of cleaved PARP, caspase-3, and caspase-7, indicative of enhanced apoptosis (Fig. 6E). Notably, the levels of other chaperone proteins remained largely unaffected, with only a minor decrease in GRP94 (Figure S5B and C). These findings establish that OLN is able to suppress tumor GRP78 expression and potentiate the onset of apoptosis in vivo.

### Oleandrin inhibits SARS-CoV-2 plaque formation by suppressing GRP78 expression

**Fig. 7:**
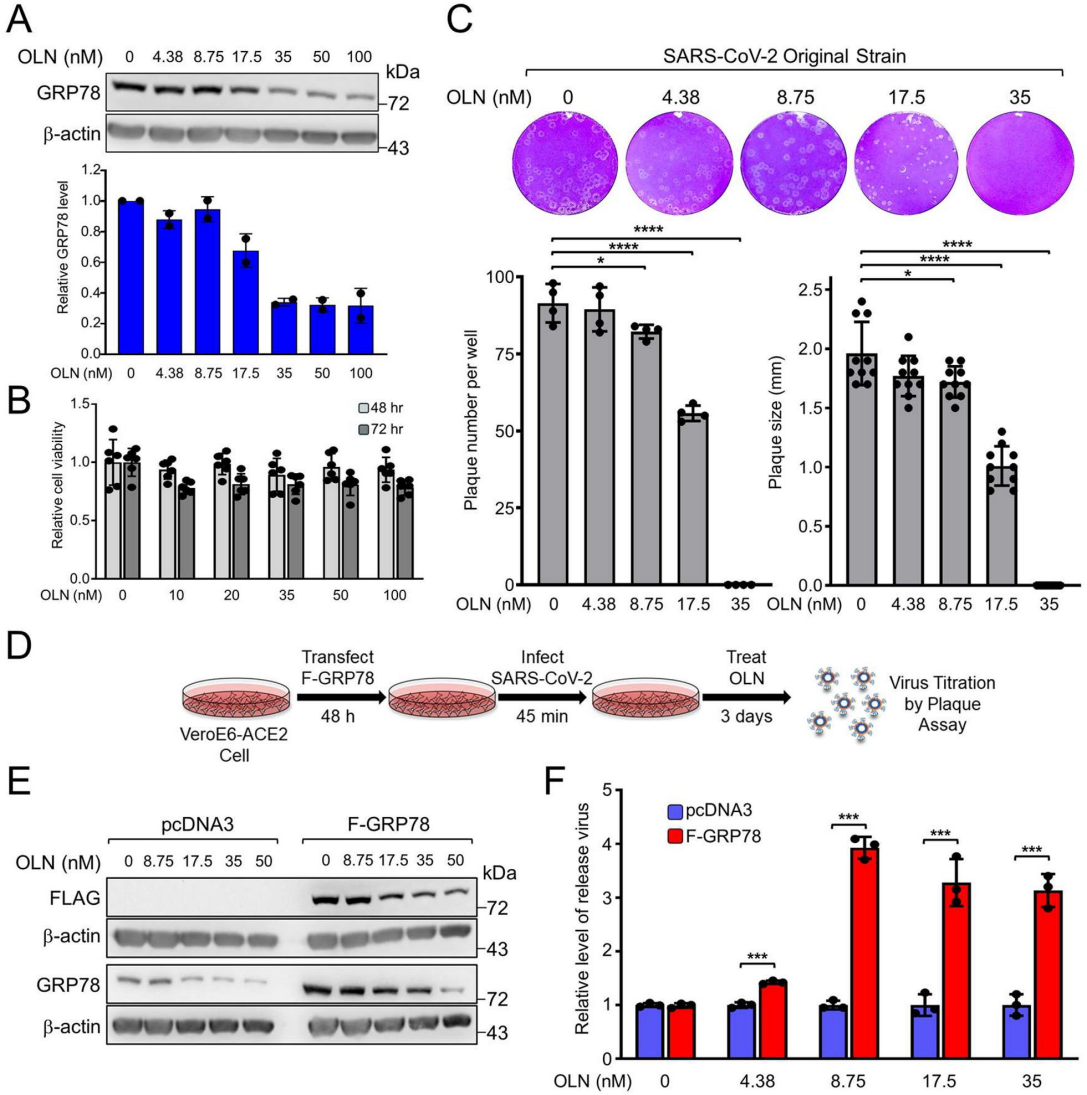
Oleandrin blocks infection by SARS-CoV-2 mediated in part via reduction of GRP78. **A** Vero E6-ACE2 cells were treated with increasing concentrations of OLN for 48 h and probed for GRP78 and β-actin levels. Quantitation of the relative GRP78 protein levels normalized to β-actin is shown in the graph below. **B** Vero E6-ACE2 cells were treated with increasing concentrations of OLN for 48 or 72 h and cell viability was measured by WST-1 assay. **C** Confluent monolayers of Vero E6-ACE2 cells in 6-well plates were infected with the wild-type SARS-CoV-2 virus (WA1 strain) and treated with increasing concentrations of OLN for 72 h. The cells were then fixed with 4% formaldehyde and stained with 0.2% crystal violet. The images are representatives of three repeats. Plaques were counted and plotted in the graph on the left (n = 4). Plaque size was measured and expressed relative to DMSO-treated control in the graph on the right (n = 10). **D** Experimental scheme to test the effect of GRP78 overexpression on SARS-CoV-2 virus production. **E** Vero E6-ACE2 cells were transfected with pcDNA3 empty vector or vector expressing FLAG-GRP78 (F-GRP78) for 24 h. The cells were then treated with increasing concentrations of OLN for 48 h. WCLs were subjected to Western blot analysis for Flag-tagged and GRP78 proteins levels with β-actin serving as loading control. **F** Vero E6-ACE2 cells were transfected with pcDNA3 empty vector or vector expressing F-GRP78 for 48 h. The cells were then infected with SARS-CoV-2 for 45 min followed by OLN treatment for 3 days. The level of viruses released into the media were quantified by plaque assay. The relative fold changes of virus release were graphed with the level of virus release in the cells transfected with the pcDNA empty vector set as 1. Data are presented as mean ± S.D. **p ≤ 0.01, ***p ≤ 0.001, ****p ≤ 0.0001 (Student’s *t* test)

Given the emerging roles of GRP78 as a critical host factor for viral entry and replication in SARS-CoV-2 infection [26, 28], we first examined the effect of OLN in Vero E6 cells overexpressing the human ACE2 receptor (Vero E6-ACE2), a cell line commonly used to study SARS-CoV-2. When the cells were treated with increasing dosage of OLN for 48 h, we observed that OLN suppressed GRP78 expression by 30% and 70% at 17.5 nM and at 35 nM respectively compared to DMSO-treated cells, without significant cytotoxicity for the host cells for up to 72 h (Fig. 7A, B and S6A). To investigate the anti-SARS-CoV-2 activity of OLN, we performed plaque reduction assay in Vero E6-ACE2 cells. The resulting plaques were quantified, and their sizes were analyzed to determine the degree of infection. As shown in Fig. 7C, OLN was effective in reducing plaque formation against SARS-CoV-2 WA1 strain and completely inhibited plaque formation at a concentration of 35 nM. Also, analyzed in Fig. 7C lower panel, OLN reduced not only the plaque number but also plaque size. We calculated OLN EC_50_ values of 20.6 ± 1.3 nM and 18 ± 1.8 nM based on plaque number and size respectively. To examine whether OLN blocks SARS-CoV-2 infection by affecting ACE2 expression, the cognate receptor for SARS-CoV-2 Spike protein, we isolated total and cell surface proteins from Vero E6-ACE2 cells with OLN and probed for ACE2 expression by Western blot analysis (Figure S6B). Interestingly, we observed that while OLN treatment increased the total ACE2 level, it did not affect the level of cell surface ACE2 suggesting that the inhibitory effects of OLN on SARS-CoV-2 infection do not involve alteration in ACE2 expression on the cell surface (Figure S6B).

To validate the role of GRP78 in SARS-CoV-2 inhibition by OLN, we transiently overexpressed F-GRP78 in Vero E6-ACE2 cells followed by SARS-CoV-2 infection, OLN treatment and virus titration (Fig. 7D). F-GRP78 was efficiently expressed in the transfected cells compared to cells transfected with the pcDNA3 empty vector, which served as negative control (Fig. 7E). In cells without OLN treatment, the total level of GRP78 was elevated by 3.7-fold following ectopic expression of F-GRP78. Upon treating the cells with increasing doses of OLN, we observed that while OLN was able to reduce the level of GRP78 in a dose-dependent manner, in cells over-expressing F-GRP78, substantial levels of GRP78 were maintained up to 35 nM of OLN (Fig. 7E). Thus, overexpression of GRP78 was able to rescue the suppressive effect of OLN on GRP78 level in these cells. Next, using the same condition, we infected SARS-CoV-2 at an MOI of 0.01 in Vero E6-ACE2 cells transiently transfected with either pcDNA3 empty vector or F-GRP78 expression vector. The virus culture was incubated in a 37 °C CO_2_ incubator for 3 days and titrated by plaque assay. As shown in Fig. 7F, the GRP78 overexpressed group showed a 3-to-fourfold higher virus titer compared to the empty vector transfected group (virus titers are summarized in Table S1). Collectively, these results demonstrate that the anti-SARS-CoV-2 activity of OLN is in part due to the suppression of GRP78 expression.

### Oleandrin is effective against SARS-CoV-2 variants and enhances current anti-COVID-19 therapies

**Fig. 8:**
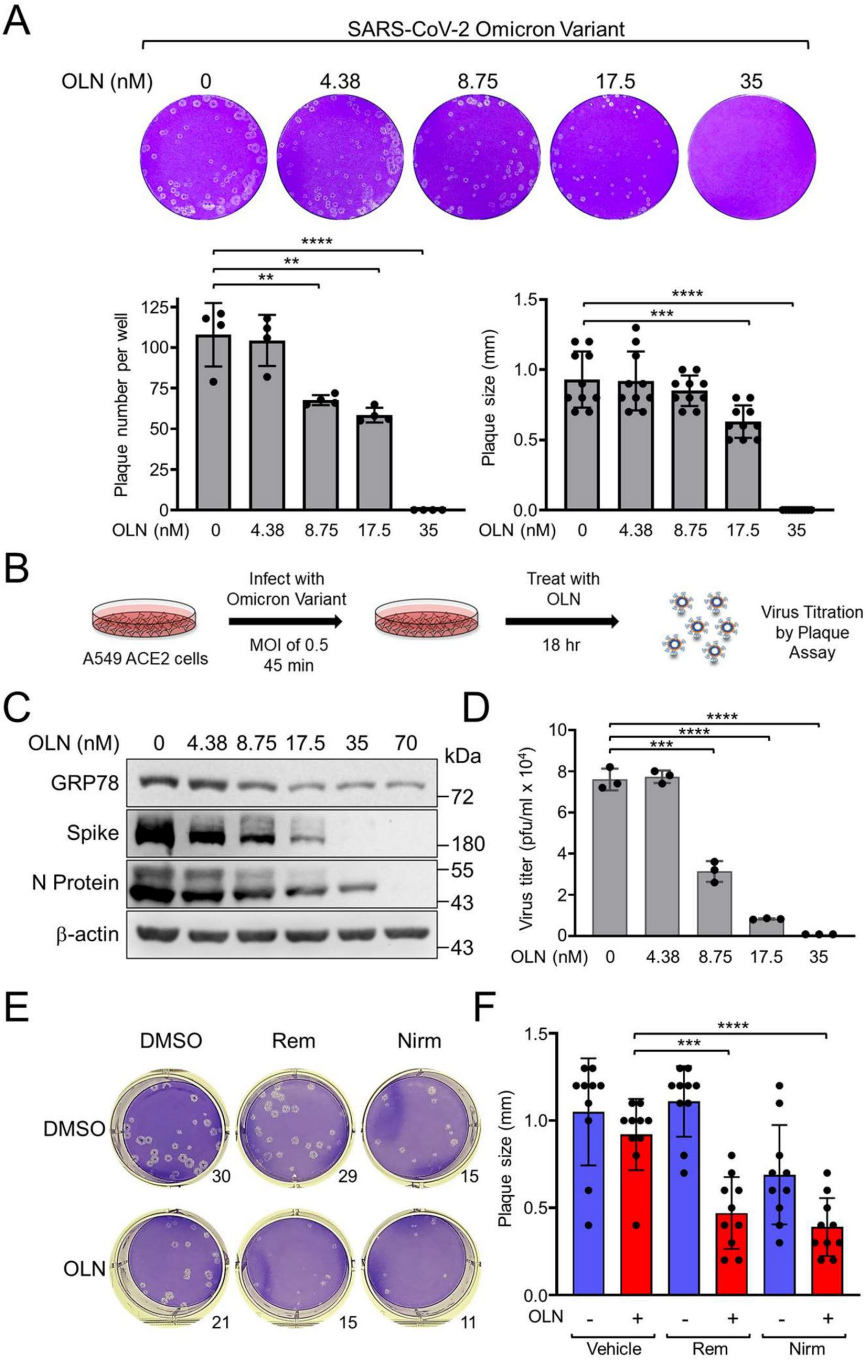
Oleandrin blocks infection by SARS-CoV-2 variants and enhances anti-viral therapies. **A** Confluent monolayers of Vero E6-ACE2 cells in 6-well plates were infected with SARS-CoV-2 Omicron variant and treated with increasing concentrations of OLN as indicated for 72 h. The cells were fixed with 4% formaldehyde and then stained with 0.2% crystal violet. The images are representatives of three repeats. Plaques were counted and plotted in the graph on the left (n = 4). Plaque size was measured and expressed relative to DMSO-treated control in the graph on the right (n = 10). **B** Experimental scheme to test the effect of OLN on viral release in human lung A549-ACE2 cells. **C** A549-ACE2 cells were infected with the Omicron variant and treated with increasing concentrations of OLN for 18 h. WCLs were subjected to Western blot analysis for GRP78, Spike, and N protein levels with β-actin serving as loading control. **D** Same as in **C** except the titers of released virus were quantified by plaque assay. **E** Confluent monolayers of Vero E6-ACE2 cells in 6-well plates were infected with SARS-CoV-2 and treated with 15 nM of OLN alone or in combination with 100 nM of Remdesivir (Rem) or 500 nM of Nirmatrelvir (Nirm) for 72 h. At the end of treatment, the cells were fixed with 4% formaldehyde and stained with 0.2% crystal violet. The number of plaques counted was shown on the lower right of the well. **F** Plaque size from **E** was measured and expressed relative to DMSO-treated control in the graph on the right (n = 10). Data are presented as mean ± S.D. ***p ≤ 0.001, ****p ≤ 0.0001 (Student’s *t* test)

The rapid antigenic drift of the SARS-CoV-2 Spike protein led to the emergence of variants such as Alpha, Beta, Gamma, Delta and other Omicron subvariants [31, 32, 49], creating the need to develop newer vaccines and antivirals against them. The merit of developing antivirals that targeting the essential host protein is that it enables us to develop broad-spectrum antivirals against viruses with similar infection mechanisms. We observed that OLN was highly effective in reducing plaque formation tested in Vero E6-ACE2 cells against the SARS-CoV-2 Omicron variant and completely inhibited plaque formation at a concentration of 35 nM (Fig. 8A). The calculated OLN EC_50_ values were 18.3 ± 3.3 nM and 21.7 ± 2.4 nM based on the plaque number and size respectively. OLN also showed high efficacy against the Beta and Delta variants and at 35 nM completely blocked plaque formation (Figure S6C). Furthermore, we performed a sequence analysis of the putative Spike protein recognition site for GRP78 across various SARS-CoV-2 strains. The PCXXXXXXNC motif within residues P479-C488 of the Spike protein, previously identified by molecular docking simulation as the GRP78 binding region [50], exhibited remarkable conservation among different strains, including the XBB.1.5.66 strain which has recently been characterized [49], as well as the more recent JN.1.11.1 strain (Figure S7). We then investigated the inhibitory effect of OLN on SARS-CoV-2 virus growth. This was achieved by infecting the Omicron variant in the human lung epithelial A549 cells expressing the hACE2 receptor (A549-ACE2), followed by OLN treatment and plaque assay to determine the virus titers (Fig. 8B). Western blot analysis revealed a OLN dose-dependent reduction in GRP78, Spike, and N protein levels without compromising the viability of these cells (Fig. 8C and S6D). Notably, viral production was dramatically reduced, even at an 8.7 nM dose of OLN, and at 17.5 nM and 35 nM, OLN treatment reduced the virus titer by up to 10- and 100-fold respectively (Fig. 8D). Thus, OLN is highly effective against the SARS-CoV-2 variants.

Currently, Remdesivir and Ritonavir-boosted Nirmatrelvir are approved by the FDA for the treatment of COVID-19. However, the usage of these drugs, especially in immunocompromised patients, raises concerns about the emergence of drug-resistant mutants with high replication fitness [51, 52]. To suppress or delay the chance of drug-resistant mutant generation, we tested whether the antiviral effect of OLN will enhance the antiviral activities of these drugs. As shown in Figs. 8E and F, compared to drugs alone, Remdesivir or Nirmatrelvir combined with 15 nM of OLN dramatically reduced both the number and size of plaques. Collectively, these results showed OLN at low nanomolar dosages can greatly enhance standard COVID-19 therapy.

## Discussion

Since its discovery in 1977, the multifunctional protein GRP78 with potent anti-apoptotic activities has emerged as a promising target for suppressing tumorigenesis and treatment resistance [8, 53]. Recent studies further revealed that GRP78 is upregulated during SARS-CoV-2 infection and acts as a pro-viral protein [28]. While advances have been made towards the development of therapeutic agents against GRP78 [8, 18, 54–57], targeting GRP78 through the repurposing of FDA-approved agents could accelerate the pace towards clinical application. Here, we identified that the cardiac glycoside OLN at nanomolar range is capable of dually suppressing stress induction of GRP78 in cancer and in SARS-CoV-2 infected cells.

Among the therapeutics targeting GRP78, while monoclonal antibodies and peptides can offer highly specific inhibition of the cell surface form of GRP78, however, they are unable to act upon intracellular GRP78 or its induction under stress. One the other hand, small molecule GRP78 inhibitors, in principle, should be able to inhibit GRP78 activity in all the intracellular compartments, as well as on the cell surface, thus simultaneously shutting down the multitude of pro-growth and pro-survival pathways regulated by GRP78, making it more difficult for cancer cells to develop resistance to anti-GRP78 therapy. Nonetheless, inhibition of GRP78 activity typically triggers ER stress, which could lead to upregulation of GRP78 [54, 55]. In contrast, CGs as a class uniformly suppress the induction of GRP78 protein across a broad spectrum of stress conditions. As cancer and virally infected cells require higher levels of GRP78 protein to overcome stress associated with tumorigenesis, therapeutic resistance, and robust viral production, this will be blocked by CGs treatment.

Among the CGs, OLN exhibits the greatest potency and functional efficacy in various cancer cells. In this study, using colorectal and breast cancer as models, we showed that not only does OLN reverse the level of GRP78 under a wide variety of stress conditions (including glucose starvation and hypoxia) back to the basal level, but the reduction in total GRP78 also resulted in decreased expression of cell surface and nuclear GRP78 under stress. Thus, OLN treatment will not only deprive cancer cells of elevated GRP78 in the ER to cope with ER stress, but also suppress its new functions as signaling and transcriptional regulators beyond the ER, impacting proliferation, survival, invasiveness, and resistance to treatment.

Mechanistically, we have identified OLN at nanomolar range is capable of suppressing stress induction of GRP78 and this strictly depends on the integrity of the Na^+^/K^+^-ATPase α3 isoform preferentially expressed in human cancer cells. This could explain in part the enhanced cytotoxicity of OLN in malignant compared to normal cells. In contrast, OLN has no activity towards GRP78 expression in mouse cells expressing Na^+^/K^+^-ATPase α3 which harbors two mutations compared to human [47]. Previous studies reported that the intracellular location and the abundance of the α3 isoform is altered in human cancer versus normal cells, thus offering an opportunity for its targeting for cancer therapy [58]. Here, we showed that OLN suppresses GRP78 induction via the α3 isoform post-transcriptionally, likely at the level of translation. In agreement, a recent study revealed that Na^+^/K^+^-ATPase α3 interacts with RNA-binding proteins and regulates protein synthesis, with knockdown of α3 reducing the protein level of chaperone protein HSP70, which is the cytosolic homolog of GRP78 [59]. Furthermore, CGs decreased the proportion of cancer stem cells and sensitized breast cancer to drug therapy [60]. It is tempting to speculate that a contributing factor of the potency of CGs in breast cancer could be due to suppression of stress induction of GRP78, as observed in this study.

Cardiac glycosides are known to affect diverse pathways in exerting their anti-tumor activities, including suppression of general protein synthesis [34–38, 46]. In this study, we determined that OLN did not suppress stress induction of *GRP78* transcription in HCT116 cells, and in the case of MDA-MB-231 cells, OLN further enhanced stress induction of *GRP78* transcripts. OLN, at the 35 nM dosage, mildly inhibited new protein synthesis in stressed and non-stressed cells. In performing polysome profiling of *GRP78* mRNA, we observed that upon OLN treatment of ER stressed cells, higher levels of *GRP78* transcripts were detected as free RNA or associated with 80S ribosomal subunit, corresponding with a decrease in its association with polysomes, consistent with previous reports that cells under stress utilize translational regulation to allow immediate and selective changes in protein levels [61]. Here, we identified that human Na^+^/K^+^-ATPase α3 is required for OLN suppression of GRP78 stress induction; however, future studies will be required to dissect out the precise mechanisms.

Interestingly, specific cancers have been reported to express microRNAs that can cooperatively function to suppress GRP78 translation and reverse chemoresistance [62]. Here, we demonstrated that in HCT116 cells, apoptosis induced by an ER stressor such as Tg as well as chemotherapeutic agents (5-FU, Irinotecan, and Oxaliplatin) was enhanced by OLN, and this could be reversed through GRP78 overexpression. Furthermore, OLN reduced the expression of GRP78 as well as the viability of human colon cancer cells resistant to an anti-EGFR targeted therapy, Cetuximab. Utilizing organoids derived from colon cancer patient tumors [63], which offer a more physiologically relevant 3D human cancer model for drug testing, we observed that OLN, in the dosages that suppressed stress induction of GRP78, lowered organoid viability under normal and hypoxic conditions. Furthermore, in an orthotopic xenograft model of MDA-MB-231, an aggressive, metastatic human breast cancer cell line, OLN treatment resulted in suppression of GRP78 expression in the tumor, associating with the onset of apoptosis and reduction in tumor load. In support, a recent study revealed that PBI-05204, an herbal extract containing OLN as an active component, suppresses glioblastoma stem cells through GRP78 inhibition and induces programmed necroptotic cell death [64]. Collectively, these studies established that OLN is capable of suppressing GRP78 expression in both in vitro and in vivo human cancer models. Regarding its safety, it has been reported that OLN while toxic to malignant cells, even at high nanomolar range did not affect the viability of peripheral blood mononuclear cells and neutrophils [65]. Nonetheless, to optimize its effectiveness, it may be best used in combination with conventional therapies, acting as a sensitizer to mitigate drug resistance.

Viruses, as obligate intracellular parasites, are reliant upon the host cell's molecular machinery for the synthesis of their genetic materials and the production of essential structural proteins critical for their replication. Functioning as a major chaperone in the ER, GRP78 plays multiple roles in the viral life cycle [27]. In addition to assisting in viral entry [26], intracellular GRP78 facilitates the proper folding of newly synthesized viral proteins. Moreover, owing to its pro-survival attributes, GRP78 sustains host cell viability, ensuring a conducive environment for viral propagation. Here we report that OLN at dosages with no harmful effects on the host cells can suppress GRP78 expression and reduce the infectivity of SARS-CoV-2, the Beta and Delta, as well as the highly transmissible Omicron variants. In this study, we performed sequence analysis and noted a high degree of sequence conservation in the Spike-GRP78 interaction site across many different variants including the more recent Omicron strains (XBB.1.5.66 and JN.1.11.1). This suggests that OLN, by targeting a conserved host factor essential for Spike binding, may retain efficacy against future SARS-CoV-2 variants. We further established that suppression of GRP78 expression contributes to its anti-COVID-19 activity. Importantly, OLN can potentiate the effect of Remdesivir and Nirmatrelvir in suppressing SARS-CoV-2 infectivity in human lung cells. The development of antivirals targeting essential host proteins not only provides the advantage of creating broad-spectrum antivirals effective against viruses with similar infection mechanisms but also allows for their combination with drugs currently used in clinics, which have different mechanisms of action. Implementing this combination approach has the potential to reduce the dosage needed for human use, ultimately contributing to a decrease or delay in the emergence of drug-resistant mutants, particularly in immune-compromised individuals.

In summary, cancer and SARS-CoV-2 upregulate GRP78 and hijack its functions at multiple cellular locations to promote tumorigenesis and viral infectivity. Thus, agents that can suppress its induction and its function at various locations could offer dual protection against cancer and COVID-19. Recently, OLN was discovered by machine learning as a highly potent senolytic compound, and it is noted that while CGs as a class has been shown to have a narrow therapeutic window regarding cardiotoxicity, OLN with its inhibitory activity at the nanomolar range, coupled with its simple structure compared to other CGs, makes it closer to a potentially non-cardiotoxic pharmacophore [66]. OLN when used in combination therapy, will be able to synergize with other therapeutic agents, thus requiring even lower dosages. In addition, recent advances in microsphere/hydrogel delivery system for OLN at the affected sites could further enhance its potency and safety [67]. Our studies provide further groundwork for the future investigation of OLN as a natural and commonly accessible compound for dual suppression of cancer and COVID-19 and other human diseases that depend on stress induction of GRP78 for their pathological progression.

## Materials and methods

### Cell lines and culture conditions

The major cell lines used in the cancer study were as follows. The HCT116 cell line was purchased from ATCC and cultured in McCoy’s 5A medium supplemented with 10% fetal bovine serum (FBS; GeminiBio, West Sacramento, CA) and 1% penicillin/streptomycin (pen/strep; Corning Inc., Glendale, AZ). The human triple-negative breast cancer cell line MDA-MB-231 and its derivative MDA-MB-231-GFP/Luc have been previously described [68] and were cultured in Dulbecco’s modified Eagle medium (DMEM, Corning Inc., Glendale, AZ) supplemented with 10% FBS and 1% pen/strep. The isogenic Cetuximab-sensitive LIM1215 (*KRAS* wildtype) and Cetuximab-resistant LIM1215R4 (*KRAS* mutant) colorectal cancer cell lines have been previously described [69] and were cultured in RPMI-1640 media (Corning Inc., Corning, NY), supplemented with 10% FBS and 1% pen/strep. Mouse embryonic fibroblast cell line (MEF), mouse acinar pancreatic cancer cell line 266–6 (Gift from Dr. Hamid M. Said, UCI) were cultured in DMEM supplemented with 10% FBS and 1% pen/strep. Human head and neck cancer cells SCC15, SCC25, and SCC351 (Gift from Dr. Vicky Yamamoto, USC) have been previously described [12] and were cultured in DMEM supplemented with 10% FBS and 1% pen/strep. The cell lines used in the survey for the prevalence of Na^+^/K^+^-ATPase α1 and α3 (A427, A549, H522, RKO, SW480, PanC1, CFPAC1, MCF7, C4-2B, SK-MEL-28, H929, and HepG2) and their culture conditions were previously described [20, 70–72].

The cell lines used in the virus study were as follows. The African green monkey kidney epithelial cell line Vero E6 expressing ACE2 (Vero E6-ACE2) was a gift from Dr. Younho Choi (University of Southern California). The human colorectal adenocarcinoma cell line Caco-2 expressing ACE2 (Caco-2-ACE2) was a gift from Drs. GuanQun Liu and Michaela Gack (Florida Research and Innovation Center). Human Lung Carcinoma Cells A549 Expressing Human Angiotensin-Converting Enzyme 2 (A549-ACE2) was obtained from the Centers for Disease Control and Prevention and obtained through BEI Resources, NIAID, NIH (NR-53821). Vero E6-ACE2 cells were cultured in DMEM supplemented with 10% FBS, 1% pen/strep and 1 µg/ml puromycin. A549-ACE2 cells were cultured in DMEM supplemented with 10% FBS, 1% pen/strep and 100 µg/ml blasticidin. All cells were maintained at 37^0^C in a humidified atmosphere of 5% CO2 and 95% air.

The cell lines were routinely tested for mycoplasma contamination.

### Compounds and treatment conditions

Lanatoside C, digoxin, bufalin, ouabain, oleandrin, chloroquine, and 3-methyladenine were purchased from Millipore Sigma (St. Louis, MO). The proteasome inhibitors MG101, MG115, and MG132 were purchased from Selleckchem (Houston, TX). The ER-stress inducers thapsigargin (Tg), tunicamycin (Tu), and 2-deoxy-D-glucose (2-DG), and the autophagy inhibitor bafilomycin A1 were purchased from Cayman Chemical (Ann Arbor, MI). Remdesivir (HY-104077) and Nirmatrelvir (HY-138687) were purchased from MedChemExpress (Monmouth Junction, NJ). All drugs were dissolved in DMSO except for 3-MA and 2-DG which were dissolved in sterile double distilled water. The final concentration of DMSO in cell culture were either 0.1% or 1%. To induce ER stress, the cells were treated with Tg at 300 nM, Tu at 1.5 μg/ml, or 2-DG at 10 mM for 24 h. In combination treatment, cardiac glycosides and the ER-stress inducers were added to the cells at the same time and incubated for 24 h. For proteasome and autophagy inhibition, cells were treated with oleandrin, Tg, and the proteasome inhibitors MG101 (10 μM), MG115 (10 μM), MG132 (10 μM) or autophagy inhibitors 3-MA (10 mM), chloroquine (20μM), and bafilomycin A1 (100 nM) at the same time and incubated for 24 h. For the drug-OLN combinational treatment study, 15 nM of OLN was treated alone or in combination with Remdesivir or Nirmatrelvir at concentrations of 100 nM and 500 nM, respectively. For all experiments, DMSO was used as vehicle control.

### Hypoxia induction and glucose starvation

For hypoxia induction, we utilized the Galaxy 48r Incubator CO_2_/O_2_/N_2_ (Eppendorf, Hamburg Germany). The chamber was set at 0.1% O_2_ and 5% CO_2_. Regular culture medium (DMEM 10% FBS 1% pen/strep) was incubated at 0.1% O_2_ and 5% CO_2_ for 48 h before the start of the experiment to decrease the oxygen concentration in the medium. This is the “equilibrated medium”. HCT116 and MDA-MB-231 cells were seed at 5 × 10^5^ cells per 6 cm dish and allowed to attached overnight. Next day, the cells were switched to the “equilibrated medium” prepared above and treated with DMSO vehicle control or 35 nM of oleandrin and incubated at 0.1% O_2_ and 5% CO_2_ for 24 h. An identical set of cells were incubated at regular culture condition (20% O_2_ and 5% CO_2_). After 24-h incubation period, cells were washed once with ice cold PBS and immediately lysed with RIPA buffer. Cell lysates were subjected to Western blot analysis to detect the proteins of interest.

For glucose starvation, HCT116 and MDA-MB-231 were seeded at 5 × 10^5^ cells per 6 cm dish and allowed to attach overnight. Next day, cells were washed twice with PBS to remove any trace of glucose and incubated in glucose-free DMEM supplemented with 1% pen/strep (Thermo Fisher Scientific, Cat# 11966025, Waltham, MA) and treated with DMSO vehicle control or 35 nM of oleandrin for 24 h. Cells were then washed with PBS and lysed with RIPA buffer. Cell lysates were subjected to Western blot analysis to detect the proteins of interest.

### Antibodies for immunoblots

The following antibodies were used in this study. Primary antibodies: mouse anti-GRP78 antibody (1:1000, BD Biosciences, San Jose, CA, 610979), rat anti-GRP94 antibody (1:1000, Enzo Life Sciences, Farmingdale, NY, SPA-851), mouse anti-HSP70 antibody (1:1000, Santa Cruz Biotechnology, Inc., Dallas, TX, sc-66048), rabbit anti-calnexin antibody (1:2000, Enzo Life Sciences, ADI-SPA-860), rabbit anti-PDI antibody (1:2000, Enzo Life Sciences, ADI-SPA-890), mouse anti-β-actin antibody (1:5000, ProteinTech, Rosemont, IL, 66009–1-Ig), mouse anti-FLAG M2 antibody (1:2000, MilliporeSigma, F1804), rabbit anti-cleaved PARP (Asp214) antibody (1:1000, Cell Signaling, Danvers, MA, #5625), rabbit anti-cleaved caspase 3 (Asp175) antibody (1:1000, Cell Signaling, #9661), rabbit anti-cleaved caspase 7 (Asp198) antibody (1:1000, Cell Signaling, #8438), mouse anti-E-cadherin antibody (1:1000, BD Biosciences, 610181), mouse anti-GAPDH antibody (1:5000, Santa Cruz Biotechnology, Inc., sc-32233), rabbit anti-Sodium/Potassium ATPase α3 antibody (1:1000, GeneTex, Inc., Irvine, CA, GTX53511), rabbit anti-Na,K-ATPase α1 (1:1000, Cell Signaling, #3010), mouse anti-Puromycin antibody (1:1000, Development Studies Hybridoma Bank, Iowa City, IA, PMY-2A4), rabbit anti-ATF4 (CREB2) antibody (1:500, Santa Cruz Biotechnology, Inc., sc-200), mouse anti-CHOP (L63F7) antibody (1:1000, Cell Signaling, #2895), rabbit anti-Phospho-eIF2α (Ser51) antibody (1:1000, Cell Signaling, #9721), mouse anti-ATF6 antibody (1:1000, IMGENEX, San Diego, CA), rabbit anti-ACE2 (1:1000; Proteintech, Rosemont, IL, 21,115–1-P), mouse-anti-Annexin A2 (1:1000, BD Biosciences, 610068). Secondary antibodies: horseradish peroxidase (HRP) conjugated goat anti-mouse (sc-2005), goat anti-rabbit (sc-2004), and goat anti-rat (sc-2006) antibodies (1:1000, Santa Cruz Biotechnology, Inc.), mouse IgG $\kappa$ binding protein conjugated to HRP (1:1000, Santa Cruz Biotechnology, Inc., sc-516102), mouse anti-rabbit IgG conjugated to HRP (1:1000, Santa Cruz Biotechnology, Inc., sc-2357), goat anti-mouse IRDye^®^ 800CW (1:1000, LI-COR Biosciences), goat anti-rabbit IRDye® 680RD (1:1000, LI-COR Biosciences).

### Plasmids

The construction of the FLAG-GRP78 expression vector has been described previously [15]. The pcDNA3 empty vector was used as control.

### Viruses

The following viral stocks were deposited by the Centers for Disease Control and Prevention and obtained through BEI Resources, NIAID, NIH: SARS-Related Coronavirus 2, Isolate USA-WA1/2020 (Original Strain) (NR-52281), SARS-Related Coronavirus 2, Isolate hCoV-19/USA/MD-HP20874/2021 (Omicron Variant, Strain B.1.1.529) (NR-56461), SARS-Related Coronavirus 2, Isolate hCoV-19/South Africa/KRISP-K005325/2020 (Beta Variant) (NR-54009) and SARS-Related Coronavirus 2, Isolate hCoV-19/USA/MD-HP05285/2021 (Delta Variant) (NR-55671).

All the viruses used in this study were propagated in Caco-2-ACE2 cells in DMEM incubated at 37 °C with 5% CO_2_ for 48 h. Supernatant was collected, passed through a 0.45 μm pore size polyethersulfone (PES) syringe filter, aliquoted and stored at -80ºC until further use. The virus titer was determined by plaque assay as previously described [73].

### Transfection of plasmids and siRNAs

Expression vectors were transfected into HCT116 cells using the BioT Transfection Reagent (Bioland Scientific, Paramount, CA) according to manufacturer’s recommendations. The cells were incubated with the transfection mix for 24 h to allow for protein expression before the addition of Tg and OLN and were incubated for a further 24 h before harvesting for immunoblot analysis.

The control siRNA (siCtrl) and siRNA targeting the Na^+^/K^+^-ATPase α3 isoform were purchased from Integrated DNA Technologies (IDT, Coralville, IA) with proprietary sequences. The siRNAs were transfected into cells using Lipofectamine™ RNAiMAX Transfection Reagent (ThermoFisher, Waltham, MA) according to manufacturer’s recommendations. The cells were incubated with the transfection mix for 24 h to allow for gene knockdown before the addition of Tg and OLN and were incubated for a further 24 h before harvesting for immunoblot analysis.

### Transfection of plasmids for viral studies, virus infection and cell harvest

Vero E6-ACE2 cells were seeded in 6-well plate (10^6^ cells/well) and cultured in DMEM supplemented with 10% FBS and 1% pen/strep and incubated at 37 °C overnight. The cells were washed once with fresh DMEM and transfected with 2 μg of either pcDNA3 empty vector or FLAG-GRP78 using BioT transfection reagent (Bioland Scientific) according to the manufacturer’s instructions. At 48 h post-transfection, the cells were washed once with fresh DMEM and infected with 0.01 MOI of virus in each well. The plates were incubated on a rocker at 37 °C for 45 min for virus adsorption. The virus inoculum was then removed and replaced by fresh DMEM containing twofold serial dilutions of OLN and placed in 37 °C incubator for 3 days. The supernatant was collected, filtered with 0.45 μm syringe filter and stored at -80ºC. The supernatant samples were subjected to plaque assay for virus titration.

A549-ACE2 cells were seeded in 6-well plates and incubated overnight. Next day, the cells were washed once with fresh DMEM and infected with 0.5 MOI of virus in each well. The plates were incubated on a rocker at 37 °C for 45 min for virus adsorption. The virus inoculum was then removed and replaced by fresh DMEM containing the indicated concentrations of OLN and placed in a 37 °C CO_2_ incubator. The cell pellets were collected and stored at − 80 °C. The cell pellet samples were lysed, and cell lysates were subjected to immunoblot analysis as described above.

### RT-qPCR

Cells in 6 cm culture dishes were washed 3 times with ice cold DPBS and scrapped with 1 ml of TRI Reagent (Millipore-Sigma). The total RNA was extracted according to manufacturer’s instructions. Total RNA was measured by a NanoDrop 1000 Machine (ThermoFisher, Waltham, MA) to determine the concentration and purity. To synthesize complementary DNA (cDNA), 1 μg of total RNA was used in qScript cDNA SuperMix First-Strand cDNA Synthesis Kit (QuantaBio, Beverly, MA) according to manufacturer’s recommendations. For RT-qPCR analysis, cDNA was amplified by the KAPA SYBR® FAST qPCR Master Mix (Roche Sequencing and Life Science, Wilmington, MA) and detected by the Stratagene MX3000P Real-Time QPCR System (Agilent, Santa Clara, CA) with the following PCR conditions (40 cycles, 15 s at 95^0^C, 15 s at 55^0^C, 30 s at 72^0^C). Melting curve analysis was performed to ascertain the specificity of the primers. One single peak was observed for each PCR product. The primers for human *GRP78* are 5’-GGTGAAAGACCCCTGACAAA-3’ and 5’-GTCAGGCGATTCTGGTCATT-3’, for human *β-actin* are 5’-TCCCTGGAGAAGAGCTACGA-3’ and 5’-AGCACTGTGTTGGCGTACAG-3’.

For XBP-1 splicing assay, 1 μl of cDNA was amplified in regular PCR with the following conditions (35 cycles, 95^0^C for 30 s, 58^0^C for 30 s, 72^0^C for 45 s). The primers for human XBP-1 are Forward: 5’-TTACGAGAAAACTCATGGC-3’ and Reverse: 5’- GGGTCCAAGTTGTCCAGAATGC-3’. PCR products were electrophoresed on 3% agarose gel and DNA bands were visualized by the ChemiDoc XRS + Imager (Bio-Rad Laboratories, Hercules, CA).

### Cell surface biotinylation and purification

Cells were seeded on 6 cm culture dishes and treated as indicated. After 24 h drug treatment, cells were washed 3 times with ice cold PBS and incubated with EZ-Link Sulfo-NHS-SS-Biotin (Thermo Scientific, Waltham, MA) dissolved in PBS at a concentration of 0.5 mg/ml for 30 min at 4^0^C with gentle agitation. The biotinylation labeling solution was removed after 30 min and the reaction was quenched by Tris–Cl buffer pH 7.4 in cold PBS. Cells were then washed 3 times with PBS and lysed with RIPA buffer supplemented with Protease and Phosphatase inhibitor (ThermoFisher, Waltham, MA). Part of the lysate was saved as whole cell lysate to measure the total level of the indicated proteins. The remaining lysate was incubated with High Capacity NeutrAvidin Agarose Beads (ThermoFisher, Waltham, MA) for 1 h at room temperature to purify the biotinylated cell surface proteins. After 1 h, the unbound fraction was removed, and the agarose beads were wash 10 times with RIPA buffer. The biotinylated cell surface proteins bound to the beads were released by addition of 50 μl of 2X SDS-PAGE sample buffer with heating at 95^0^C for 5 min and analyzed by Western blot.

### Immunoblot analysis

Whole cell lysates were prepared by scrapping cells from 6 or 10 cm cell culture dishes with ice cold RIPA buffer (50 nM Tris–HCl, 150 nM NaCl, 1% NP-40, 0.5% sodium deoxycholate, and 0.1% Sodium dodecyl sulfate) supplemental with Protease and Phosphatase inhibitor cocktail (ThermoFisher, Waltham, MA). The crude lysates were incubated on ice for 30 min followed by centrifugation at 13,000 RPM at 4^0^C for 15 min. The clarified lysate solution containing soluble proteins was transferred to a new tube and mixed with 6X SDS sample buffer (0.375 M Tris pH 6.8, 12% SDS, 60% Glycerol, 0.6 M β-mercaptoethanol, 0.06% bromophenol blue). The protein samples were then heated at 95^0^C for 5 min to denature all proteins. Protein samples were electrophoresed through 8%, 10%, or 12% SDS-PAGE gels and transferred to supported nitrocellulose membrane (Bio-Rad Laboratories, Hercules, CA). Immobilized proteins on the membrane were blocked with 5% non-fat dry milk in TBST solution and incubated with primary antibodies either for 2 h at room temperature or overnight at 4^0^C followed by 2 h incubation in secondary antibodies at room temperature. Protein bands were developed by enhanced luminol-based chemiluminescent substrates (ThermoFisher, Waltham, MA) and detected by the ChemiDoc XRS + Imager (Bio-Rad Laboratories, Hercules, CA) or the LI-COR Odyssey 9120 Imaging System (LI-COR Biosciences, Lincoln, NE). Image analysis and quantitation of the band intensity were performed using the Image Lab Software Version 4.0.1 build 6 (Bio-Rad Laboratories) or Odyssey V3.0 Image Analysis software (LI-COR Biosciences).

### Immunofluorescence

Cells were seeded on Millicell EZ SLIDE (MilliporeSigma, PEZGS0816) and allowed to attach overnight. Next day, the cells were treated with drugs as indicated for 24 h. Then the media was removed, and the cells were washed with PBS three times and fixed with 4% paraformaldehyde (PFA) and permeabilized in 0.02% Triton X-100 for 10 min at room temperature. For staining of cell surface GRP78, the cells were fixed with 4% PFA but not permeabilized. After incubation with blocking buffer (5% BSA, 0.1% Tween-20, PBS) for 1 h, cells were incubated at 4 °C overnight with primary antibodies diluted in PBST in a humidified chamber at 4 °C. Primary antibody: mouse anti-GRP78 (1:500, MAb159, Gift from Dr. Parkash Gill, USC). Cells were washed three times with PBS and were incubated with Alexa Fluor-conjugated secondary antibodies for 30 min at room temperature, followed by three more washes with PBS. Secondary antibody: Alexa Fluor 488 goat anti-mouse antibody (1:500, Thermo-Fisher Scientific, #A-11001). Cells were mounted with VECTASHIELD Antifade Mounting Medium with DAPI (Vector Laboratories, Inc., #H1200). Cell images were acquired with Leica SP8 LIGHTNING Confocal Microscope using a 63 × oil objective.

### Immunohistochemistry

Immunostaining of paraffin-embedded tumor tissue sections was performed as described previously [74]. Tumor tissue sections were incubated at 4 °C overnight with primary antibodies. The antibodies used were: GRP78 (1:500, Abcam, Cambridge, MA, #ab108613). The immunostaining was visualized using the SignalStain Boost IHC Detection Reagent (HRP, Rabbit) (Cell Signaling, Danvers, MA, #8114) as per manufacturer’s protocol.

### Analysis of polysomal-associated *GRP78* mRNA

Sucrose solution was prepared in polysome extraction buffer (10 mM HEPES, 100 mM KCl, 5 mM MgCl_2_, pH 7.4, 100 μg/ml cycloheximide, 5 mM DTT). Sucrose gradients were prepared in SW41 ultracentrifuge tubes by mixing 15% and 45% sucrose solutions using a BioComp Gradient Master 108, according to the manufacturer’s instructions. Cells were lysed in polysome extraction buffer with added 1% Triton-X and RNAase out. Equal amounts of supernatants were loaded on top of the gradients and then centrifuged at 37,000 RPM at 4 °C for 2 h in an SW41 rotor of an Optima XPN 80 ultracentrifuge. Gradients were fractionated with a speed of 800 μl/min using a Biocomp piston gradient fractionator, which recorded the OD254nm. Fractions corresponding to 60 s intervals were collected and RNA was isolated from individual fractions using TRIzol LS Reagent (no. 10296010, Invitrogen), spiked with Firefly Luciferase (FLuc) control RNA (no L4561, Promega) as internal control. FLuc was diluted in TRIzol LS before addition to all fractions in a concentration of 0.001 μg/ml per sample. RNA quality was checked by NanoDrop and running of RNA in 1% agarose gels. These gels also show ribosomal RNA to determine monosome fractions. A fixed volume of RNA (5 μl) from each fraction was reverse transcribed to cDNA using a cDNA Reverse Transcription kit (no. 4387406, Thermo Fisher Scientific). cDNA samples were diluted 1:40, and 2 μl of template was used for qPCR (40 cycles: 15 s at 95 °C, 1 min at 60 °C) with Applied Biosystems TaqMan Assays. The probe used for GRP78/HSPA5 is Hs00946350_g1 (Catalogue #4351372) and for luciferase Mr03987587-mr (Catalogue #4331182). RNA levels were quantified, normalized to internal FLuc as control, summed across all fractions, analyzed, and presented as percentages of this total.

### Puromycin labeling assay

HEK293T cells were seeded on 6 cm dishes and allowed to attached overnight. Next day, the cells were treated with OLN (0–100 nM) alone or in combination with Tg (300 nM) for 8 h followed by labeling with puromycin (10 μg/ml) for 30 min. Cells were then lysed and whole cell lysates were subjected to Western blot analysis for puromycin-labeled proteins.

### Cell viability assay

Vero E6-ACE2 cells or A549-ACE2 cells were seeded at a density of 1 × 10^4^ cells per well in a 96-well flat bottom plate with DMEM supplemented with 10% FBS and 1% pen/strep. After overnight culture in 37 °C incubator, the medium was removed and replaced with serum-free DMEM and indicated concentrations of OLN at a final DMSO concentration of 0.1%. As a non-treatment control, 0.1% DMSO was used throughout the assay. Cell viability was measured at 48 and 72 h post-treatment using the (4-[3-(4-Iodophenyl)-2-(4-nitro-phenyl)-2H-5-tetrazolio]-1,3-benzene sulfonate) WST-1 cell proliferation assay kit (Takara Bio USA, Inc., San Jose, CA) according to the manufacturer’s recommendation. Colorimetric quantitation was achieved using a Model 680 Microplate Reader (Bio-Rad Laboratories, Hercules, CA) at a wavelength of 450 nm and subtracted by a reference wavelength of 650 nm. The background absorbance of blank media (DMEM 1% pen/strep) was also measured and subtracted from the sample reading.

### Colony formation assay

HCT116 cells were seeded at a density of 1 × 10^3^ cells per well in 6-well plates. Following drugs treatment for 24 h, the media was replaced with fresh media and the cells were allowed to grow for 2 weeks with frequent media change every 3 days. After the 2-week period, cells were washed with PBS, fixed in 100% methanol for 15 min at room temperature followed by Coomassie Blue staining for 30 min to visualize surviving colonies. The number of surviving colonies were counted by ImageJ software (U.S. National Institutes of Health, Bethesda, MD).

### Plaque reduction assay

Evaluating the antiviral activities of OLN, Remdesivir and Nirmatrelvir was done by plaque reduction assay as described previously with minor modifications [75]. Briefly, confluent monolayers of Vero E6-ACE2 cells in 6-well plates were washed once with DMEM and infected with approximately 30 to 100 plaque forming units (PFUs) of virus in each well. Virus-free DMEM was used for mock infections. The plates were incubated on a rocker at 37 °C for 45 min for virus adsorption. The virus inoculum was then removed and replaced by overlay media (DMEM containing 1% low-melting agarose without serum) containing desired concentrations of either OLN, Remdesivir or Nirmatrelvir and placed in 5% CO_2_ incubator at 37 °C until plaques could be visualized under light. The cells were fixed with 4% formaldehyde solution for at least 30 min and the overlaid agarose was removed and stained with 0.2% (w/v) crystal violet solution. The plaques were counted by visual examination and the size of the plaques were measured by scale loupe.

#### Patient-derived colon cancer organoids culture

For organoid generation, metastatic colon tumor tissue localized to liver was received from a consented patient following Institutional Review Board (IRB) approval at the Norris Comprehensive Cancer Center of the University of Southern California, Los Angeles, California. Patient‐derived metastatic colorectal tumor organoids (PDOs) were developed as previously described [63]. In brief, tissue was minced, digested with collagenase (Millipore, Temecula, CA), hyaluronidase (MP Biomedicals, Solon, OH) and L-Y27632 (Sigma, St. Louis, MO), cells were passed through a 100 μm strainer, washed with media, resuspended in BME (R&D System, Minneapolis, MN) and cultured with colon tumor organoid (CTO) media: ADMEM/F12 (ThermoFisher Scientific, Waltham, MA), 10% FBS (Gemini Bio, Sacramento, CA, USA), 1% P/S (Gemini Bio, Sacramento, CA), 1 × GlutaMax (ThermoFisher Scientific, Waltham, MA), 1 × HEPES (ThermoFisher Scientific), 1 × B-27 (ThermoFisher Scientific), 100 ng/ml noggin (Tonbo Biosciences, San Diego, CA), 1 × N-2 (ThermoFisher Scientific), 10 mM nicotinamide (Sigma-Aldrich), 1 mM N-acetylcysteine (Sigma-Aldrich), 500 nM A-83–01 (MilliporeSigma, Burlington, MA), 50 ng/ml EGF (Life Technologies, Grand Island, NY), and 10 μM SB202190 (Sigma-Aldrich).

### OLN drug response curve and cell harvest

For cell line-based drug treatment studies, LIM1215 WT and R4 cells were seeded at 1.5 × 10^3^ cells per well in 96-well black-wall, clear-bottom plates (Revvity, Waltham, MA). The following day, cells were dosed with OLN at concentrations ranging from 1 to 500 nM. After 3 days of OLN treatment, cells were stained with 5 μg/ml Hoechst 33342 (Life Technologies, Grand Island, NY) and 5 µg/ml propidium iodide (Life Technologies) prior to imaging to identify live/dead cells on the Operetta High Content Screening (HCS) system (Revvity, Waltham, MA). Cells were then segmented based on Hoechst-stained nuclei and classified as alive or dead based on propidium iodide intensity using Harmony software (Revvity, Waltham, WA) [76].

For PDO-based drug treatment studies, PDOs were fragmented to single cells with TrypLE Express (ThermoFisher Scientific) and filtered through a 40 μm strainer to remove aggregates, seeded on a 96-well white-wall, clear-bottom plate (Corning, Corning, NY) at a concentration of 2.5 × 10^3^ cells per well, and topped with 100 μl of CTO media. PDOs were incubated for 4 days and then treated with OLN at different concentrations from 5 to 400 nM for 3 days at normoxic (21% oxygen) and hypoxic (1% oxygen) conditions (O_2_ level maintained using BioSpherix hypoxia chamber glovebox and incubator). On day 3 of treatment, 100 μl of CellTiter-Glo 3D solution (Promega, WI) was added to each well, and cell viability was analyzed by measuring luminescence with a BioTek Synergy Neo2 Multi-Mode Reader (Agilent Technologies, Santa Clara, CA). Images of PDOs were also acquired after 3 days of treatment using the Operetta CLS HCS (Revvity, Waltham, WA) and displayed with maximum projections of 24 z-stacks ranging from 10–470 μm in increments of 20 μm.

LIM1215 WT and R4 cells cultured in 10 cm dishes were treated with OLN at different concentrations from 5 to 50 nM for 3 days before being harvested, centrifuged, and stored as snap frozen pellets. PDOs were cultured in normoxia (21% oxygen) and hypoxia (1% oxygen) conditions for 3 days before being harvested, removed from BME with Gentle Cell Dissociation Reagent (StemCell Technologies, Cambridge, MA), centrifuged, and stored as snap frozen pellets. Cell pellets were subjected to immunoblot analysis as mentioned above.

### Tumor xenograft model

1 × 10^6^ MDA-MB-231-GFP/Luc cells were orthotopically injected into the #4 fat pad of 6- to 8-week-old female NSG mice (5 mice in each group) (Jackson Laboratory, stock #005557). One week after implantation of tumor, the mice were treated with vehicle control or OLN at a concentration of 0.3 mg/kg by I.P. injection every other day excluding weekend for a 2-week period. At the end of the treatment period, the tumors were collected for Western blot and immunohistochemical analysis.

### TCGA analysis

GRP78 gene expression analysis in human normal colon and breast and colorectal adenocarcinoma and breast cancer tissues was obtained from the Gene Expression Profiling Interactive Analysis 2 (GEPIA2) [77]. The “normal tissues” included the matched adjacent normal tissues from the same patients in the TCGA database in addition to normal tissue samples from unrelated donors from the Genotype-Tissue Expression (GTEx) database [78].

### Statistical analysis

All pairwise comparisons were analyzed by unpaired 2-tailed Student’s *t* test using Microsoft Excel. All graphs were produced with GraphPad Prism version 10.0 (GraphPad Software, San Diego, CA). Data were presented as means ± Standard Deviation (S.D.). A *p*-value of ≤ 0.05 is signified by *, *p*-value of ≤ 0.01 by **, *p*-value of ≤ 0.001 by ***, and *p*-value of ≤ 0.0001 by ****, n.s. denotes not significant.

## Supplementary Information

Additional file 1: **Figure S1.** Comparative analysis of GRP78 mRNA expression in human colon and breast tissues. (A) GRP78 mRNA expression in human normal colon (n=349) and colon adenocarcinoma tumor tissues (n=275) from TCGA and GTEx databases. Data analysis was performed by the Gene Expression Profiling Interactive Analysis 2 (GEPIA2) tool. (B) Same as in (A) except the colon adenocarcinoma tumor tissues are divided into subtypes. (C) Same as in (A) except the comparison is made between human normal (n=291) and breast cancer tissues (n=810). (D) Same as in (C) except the breast cancer tissues are divided into subtypes. * p≤ 0.05 (Student's *t* test). **Figure S2.** Cardiac glycosides inhibit stress-induction of GRP78 protein in a dose-dependent manner without affecting other chaperones. (A) HCT116 cells were treated with LanC or OLN (from 10 nM to 100 nM) alone or in combination with Tg (300 nM) for 24 hr. WCLs were subjected to Western blot analysis for GRP78 protein level with β-actin serving as loading control. Quantitation of the relative levels of GRP78 normalized to β-actin are shown in the graphs below. (B) Same as in (A) except HT-29 cells were treated with OLN (from 10 nM to 100 nM) alone or in combination with Tg (300 nM) for 24 hr. (C) HCT116 cells were treated with OLN (from 10 nM to 100 nM) alone or in combination with Tg (300 nM) for 24 hr. WCLs were subjected to Western blot analysis for GRP94, HSP70, calnexin, and PDI protein levels with β-actin serving as loading control. (D) Same as in (C) except HT-29 cells were used. Data are presented as mean ± S.D. **Figure S3.** Lack of suppressive effect on GRP78 stress induction by cardiac glycosides in murine cells and OLN does not affect GRP78 transcript level in α3 knockdown cells treated with Tg. (A) Mouse embryonic fibroblasts (MEFs) or mouse acinar pancreatic cancer cells 266-6 were treated with 1 μM of lanatoside C, digoxin, ouabain, bufalin or 35 nM of oleandrin (OLN) alone or in combination with Tg (300 nM) for 24 hr. Whole cell lysates (WCLs) were subjected to Western blot analysis for GRP78 protein level with β-actin serving as loading control. (B) HCT116 cells were transfected with control siRNA (siCtrl) or siRNA targeting the NKA α3 isoform (siα3) for 24 hr. The cells were then treated with OLN (35 nM) alone or in combination with Tg (300 nM) for an additional 24 hr. Total RNA was extracted and RT-qPCR was performed to measure GRP78 mRNA levels with β-actin mRNA serving as loading control. Data are presented as mean ± S.D. **Figure S4.** Effect of oleandrin on the UPR pathways. HCT116 cells were non-treated (DMSO) or treated with OLN (35 or 50 nM) alone or in combination with Tg (300 nM) for 24 hr and analyzed for the UPR. For the PERK pathway, the protein levels of the phosphorylated form of eIF2α at Serine 51 (p-eIF2α), ATF4 and CHOP were measured by Western blot with β-actin serving as loading control. For the IRE1 pathway, the splicing of the XBP-1 mRNA transcript was analyzed by PCR with β -actin mRNA serving as loading control. The unspliced transcript is denoted with (u) and the spliced variant is denoted with (s). For the ATF6 pathway, ATF6 protein levels were measured by Western blot. The full-length ATF6 protein is denoted with (FL), and the cleaved version is denoted with (C). **Figure S5.** Oleandrin reduces GRP78 protein level in MDA-MB-231-GFP/Luc xenograft tumor tissues without majorly affecting other chaperones. (A) Immunohistochemical staining of the xenograft tumor tissues for GRP78 protein level (Scale bar, 100 μm). (B) Western blot analysis of xenograft tumor tissues for GRP94, HSP70, calnexin, and PDI protein levels with β-actin serving as loading control. (C) Quantitation of the relative protein levels normalized to β-actin in (B). Data are presented as mean ± S.D. *p≤ 0.05, n.s. denotes not significant. (Student’s t test). **Figure S6.** Oleandrin blocks infection by SARS-CoV-2 variants without impacting cell viability and does not affect cell surface ACE2 expression. (A) Confluent monolayers of Vero E6-ACE2 cells were treated with DMSO or increasing concentrations of OLN for 72 hr. Brightfield microscopy images of the cells were taken (Scale bar, 100 μm). (B) Experimental scheme to purify and detect cell surface proteins (Left). Vero E6-ACE2 cells were treated with DMSO or OLN (35 nM) for 48 hr. Whole cell lysate (WCL) and cell surface fractions were subjected to Western blot analysis for GRP78 and ACE2 protein level with GAPDH and Annexin A2 (ANXA2) serving as loading controls for total and cell surface proteins respectively. (C) Confluent monolayers of Vero E6-ACE2 cells in 6-well plates were infected with SARS-CoV-2 Beta or Delta variants and treated with increasing concentrations of OLN as indicated for 72 hr. At the end of treatment, the cells were fixed with 4% paraformaldehyde and stained with 0.2% crystal violet. Plaques were counted and plotted as percentage relative to the untreated control in the graph on the left and their size was measured and expressed relative to DMSO-treated control in the graph on the right. (D) A549-ACE2 cells were treated with increasing concentrations of OLN for 48 or 72 hr and cell viability was measure by WST-1 assay. Data are presented as mean ± S.D. **Figure S7.** Sequence alignment of the putative Spike protein recognition site for GRP78 among different variants of SARS-CoV-2. The amino acid sequences of Spike protein of different variants of SARS-CoV-2 were aligned. The putative Spike recognition site for GRP78 from residues P479-C488 with the highly conserved PCXXXXXXNC sequence is highlighted.

Additional file 2: **Table S1.** GRP78 overexpression rescues virus release of SARS-CoV-2 under oleandrin treatment. Vero E6-ACE2 cells were transfected with pcDNA3 empty vector or FLAG-GRP78 for 48 hr. The cells were then infected with SARS-CoV-2 for 45 min followed by OLN treatments for 3 days. The titers of virus released into the media were quantified by plaque assay.

## Data Availability

All data generated and analyzed during this study are included in this published article and its supplementary information files.

## Abbreviations

GRP78: 78-kilodalton glucose-regulated protein
ER: Endoplasmic reticulum
CGs: Cardiac glycosides
COVID-19: Coronavirus disease 2019
SARS-CoV-2: Severe acute respiratory syndrome coronavirus 2
OLN: Oleandrin
LanC: Lanatoside C
UPR: Unfolded protein response
csGRP78: Cell surface GRP78
nuGRP78: Nuclear GRP78
ACE2: Angiotensin-converting enzyme 2
TCGA: The Cancer Genome Atlas
Tg: Thapsigargin
Tu: Tunicamycin
2-DG: 2-Deoxy-d-glucose
NKA: Na^+^/K^+^-ATPase
PDO: Patient-derived colon cancer organoids
MOI: Multiplicity of infection
WCL: Whole-cell lysate
MEF: Mouse embryonic fibroblast
