# Supplementary material for: Targeting stress induction of GRP78 by cardiac glycoside oleandrin dually suppresses cancer and COVID-19
Source: Cell Biosci. 2024 Sep 6;14:115. doi: 10.1186/s13578-024-01297-3 (PMC11378597; doi:10.1186/s13578-024-01297-3)
Supplement: Supplementary file 1 — Additional file 1: Figure S1. Comparative analysis of GRP78 mRNA expression in human colon and breast tissues. (A) GRP78 mRNA expression in human normal colon (n=349) and colon adenocarcinoma tumor tissues (n=275) from TCGA and GTEx databases. Data analysis was performed by the Gene Expression Profiling Interactive Analysis 2 (GEPIA2) tool. (B) Same as in (A) except the colon adenocarcinoma tumor tissues are divided into subtypes. (C) Same as in (A) except the comparison is made between human normal (n=291) and breast cancer tissues (n=810). (D) Same as in (C) except the breast cancer tissues are divided into subtypes. * p≤ 0.05 (Student's t test). Figure S2. Cardiac glycosides inhibit stress-induction of GRP78 protein in a dose-dependent manner without affecting other chaperones. (A) HCT116 cells were treated with LanC or OLN (from 10 nM to 100 nM) alone or in combination with Tg (300 nM) for 24 hr. WCLs were subjected to Western blot analysis for GRP78 protein level with β-actin serving as loading control. Quantitation of the relative levels of GRP78 normalized to β-actin are shown in the graphs below. (B) Same as in (A) except HT-29 cells were treated with OLN (from 10 nM to 100 nM) alone or in combination with Tg (300 nM) for 24 hr. (C) HCT116 cells were treated with OLN (from 10 nM to 100 nM) alone or in combination with Tg (300 nM) for 24 hr. WCLs were subjected to Western blot analysis for GRP94, HSP70, calnexin, and PDI protein levels with β-actin serving as loading control. (D) Same as in (C) except HT-29 cells were used. Data are presented as mean ± S.D. Figure S3. Lack of suppressive effect on GRP78 stress induction by cardiac glycosides in murine cells and OLN does not affect GRP78 transcript level in α3 knockdown cells treated with Tg. (A) Mouse embryonic fibroblasts (MEFs) or mouse acinar pancreatic cancer cells 266-6 were treated with 1 μM of lanatoside C, digoxin, ouabain, bufalin or 35 nM of oleandrin (OLN) alone or in combination with Tg (300 nM) f [file 13578_2024_1297_MOESM1_ESM.pdf]

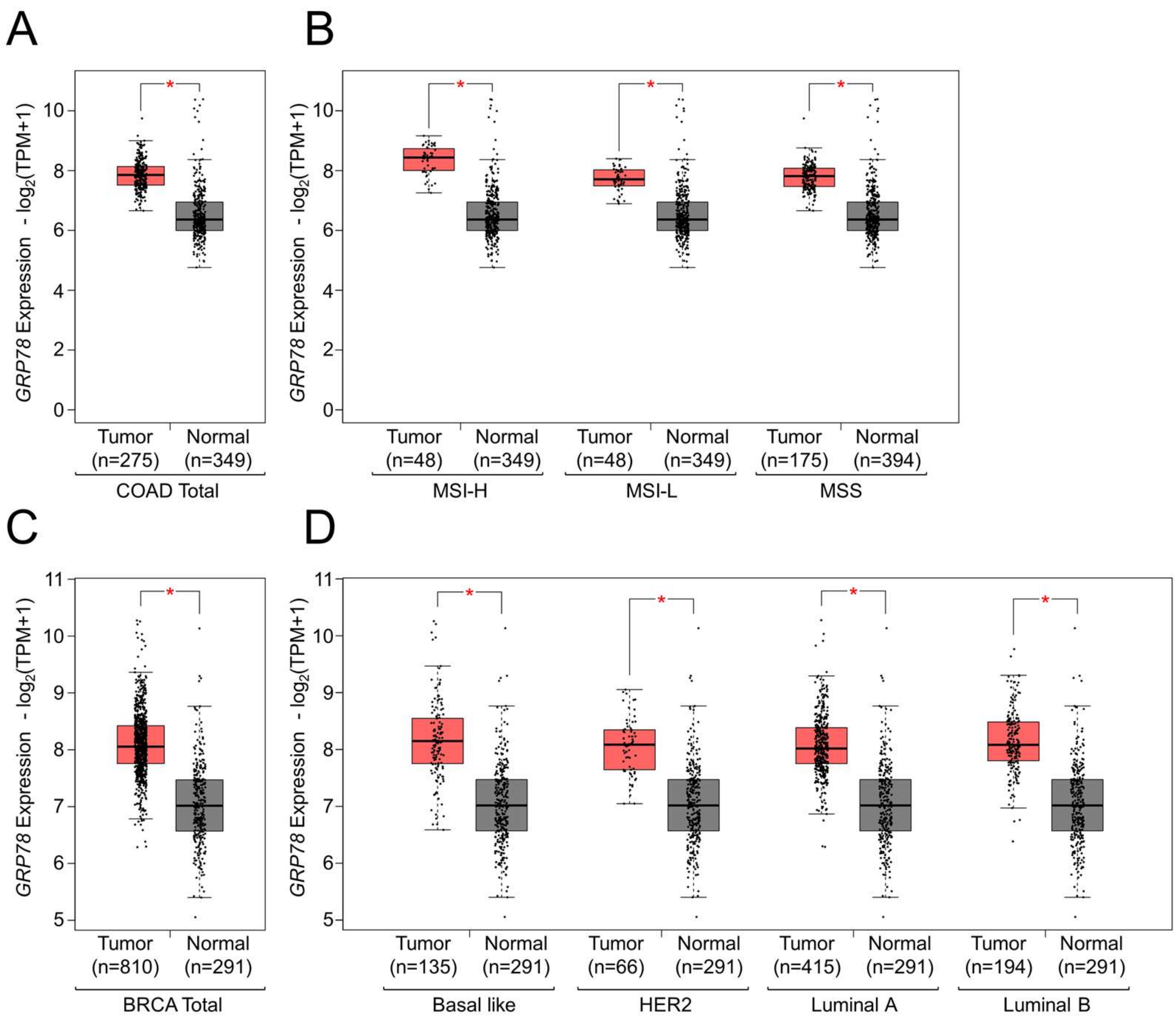
A
B
C
D
GRP78 Expression - log2(TPM+1)
Tumor (n=275)
Normal (n=349)
COAD Total
Tumor (n=48)
Normal (n=349)
MSI-H
Tumor (n=48)
Normal (n=349)
MSI-L
Tumor (n=175)
Normal (n=394)
MSS
Tumor (n=810)
Normal (n=291)
BRCA Total
Tumor (n=135)
Normal (n=291)
Basal like
Tumor (n=66)
Normal (n=291)
HER2
Tumor (n=415)
Normal (n=291)
Luminal A
Tumor (n=194)
Normal (n=291)
Luminal B

Figure S1

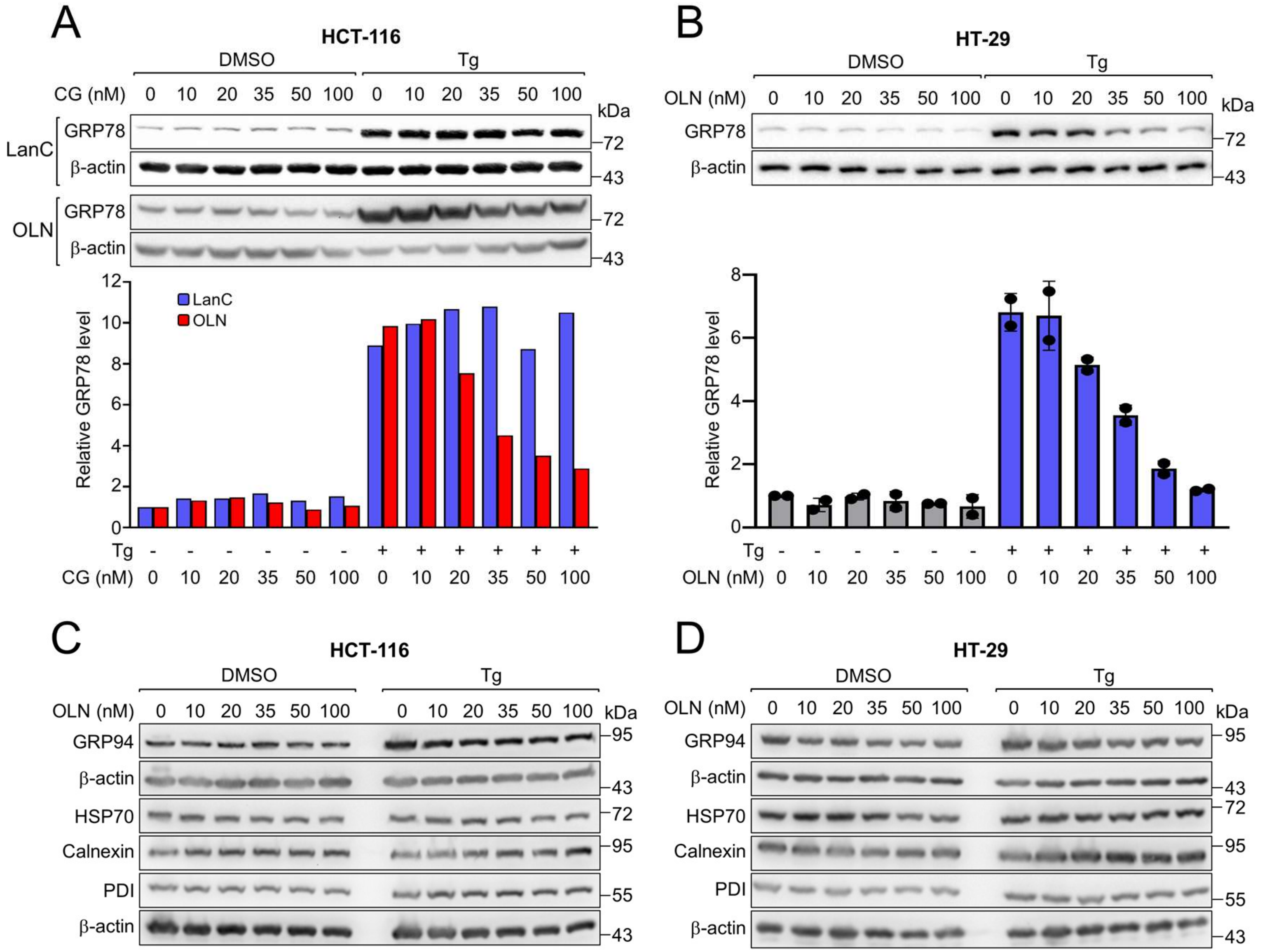
A
HCT-116
DMSO
Tg
CG (nM) 0 10 20 35 50 100 0 10 20 35 50 100
kDa
LanC
GRP78
β-actin
OLN
GRP78
β-actin
72
43
72
43
LanC
OLN
Relative GRP78 level
0 2 4 6 8 10 12
Tg - - - - - - + + + + + +
CG (nM) 0 10 20 35 50 100 0 10 20 35 50 100
B
HT-29
DMSO
Tg
OLN (nM) 0 10 20 35 50 100 0 10 20 35 50 100
kDa
GRP78
β-actin
72
43
Relative GRP78 level
0 2 4 6 8
Tg - - - - - - + + + + + +
OLN (nM) 0 10 20 35 50 100 0 10 20 35 50 100
C
HCT-116
DMSO
Tg
OLN (nM) 0 10 20 35 50 100 0 10 20 35 50 100
kDa
GRP94
β-actin
HSP70
Calnexin
PDI
β-actin
95
43
72
95
55
43
D
HT-29
DMSO
Tg
OLN (nM) 0 10 20 35 50 100 0 10 20 35 50 100
kDa
GRP94
β-actin
HSP70
Calnexin
PDI
β-actin
95
43
72
95
55
43

Figure S2

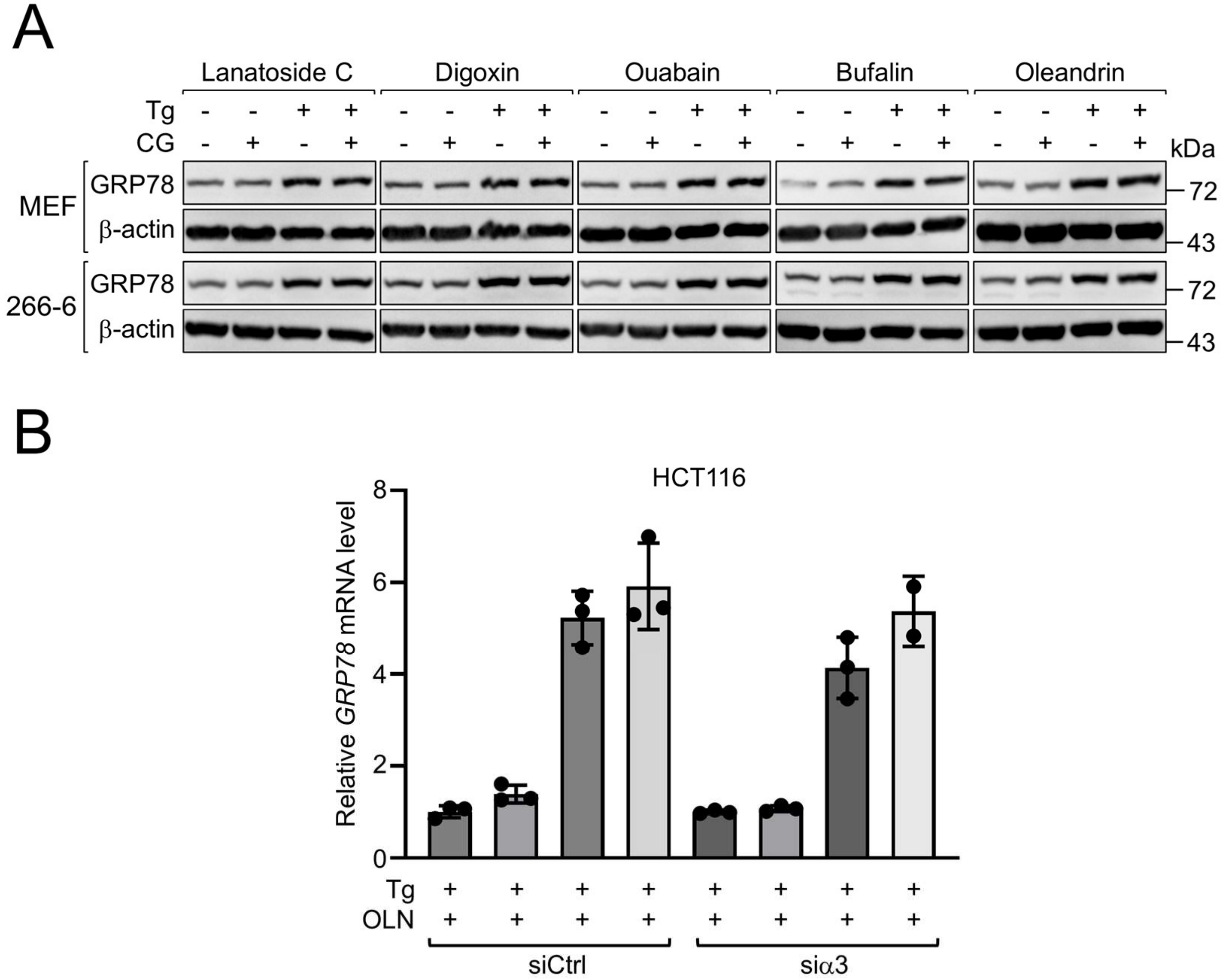
A
Lanatoside C
Digoxin
Ouabain
Bufalin
Oleandrin
Tg
CG
kDa
MEF
GRP78
β-actin
266-6
GRP78
β-actin
72
43
72
43
B
HCT116
Relative GRP78 mRNA level
8
6
4
2
0
Tg
OLN
siCtrl
siα3

Figure S3

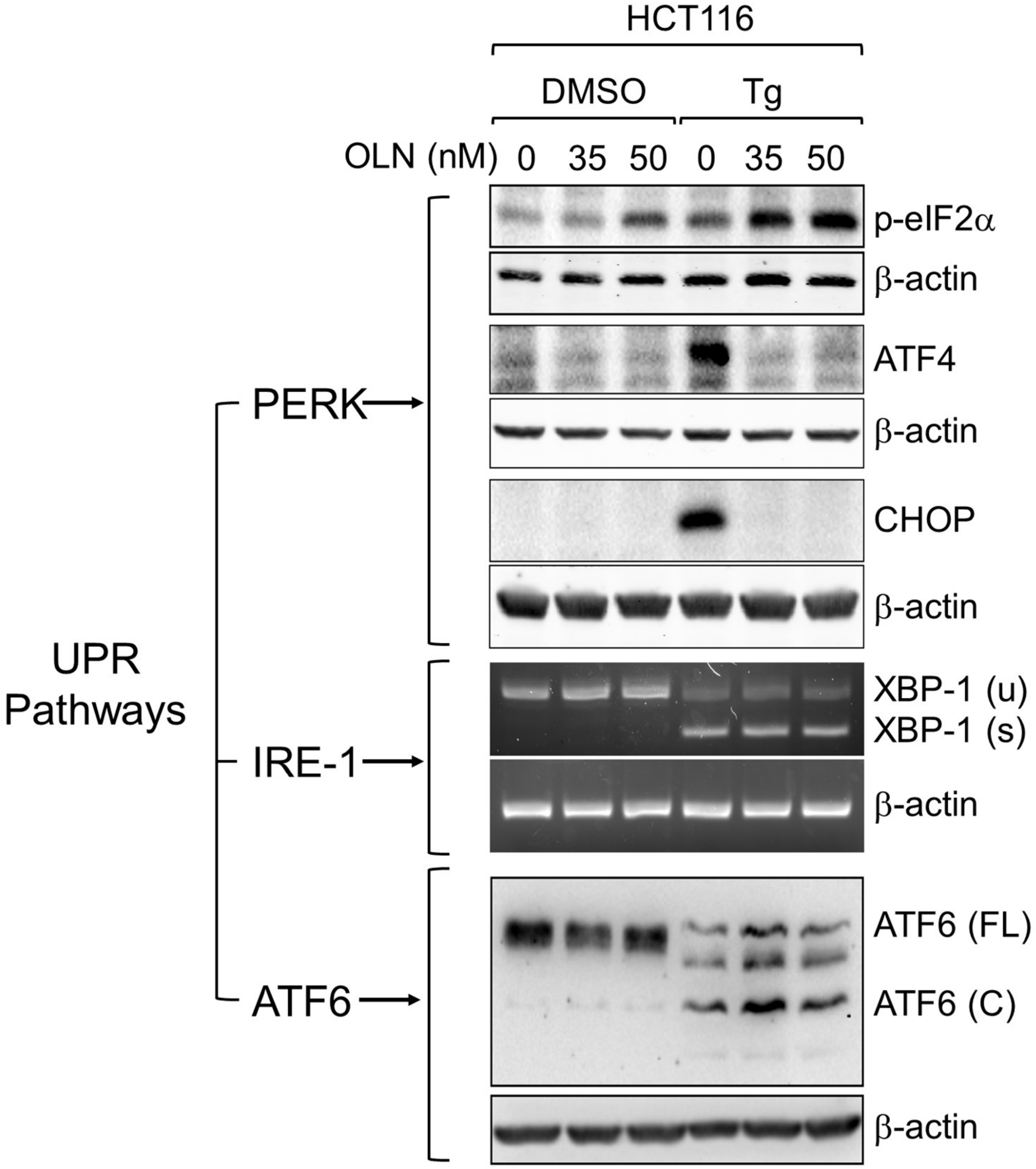
HCT116
DMSO
Tg
OLN (nM) 0 35 50 0 35 50
p-eIF2α
β-actin
ATF4
β-actin
CHOP
β-actin
XBP-1 (u)
XBP-1 (s)
β-actin
ATF6 (FL)
ATF6 (C)
β-actin
UPR Pathways
PERK
IRE-1
ATF6

Figure S4

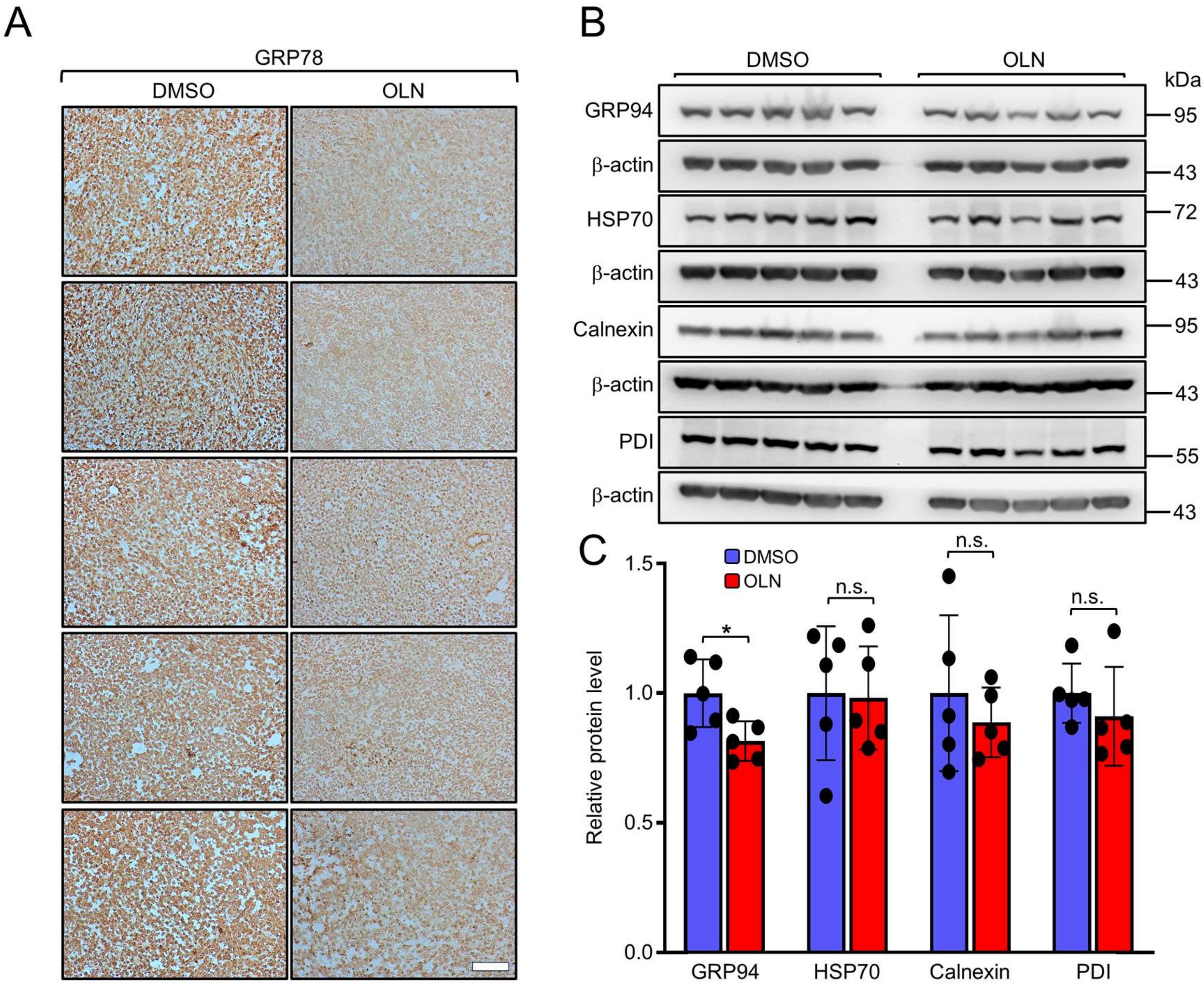
A
GRP78
DMSO
OLN
B
DMSO
OLN
kDa
GRP94
β-actin
HSP70
β-actin
Calnexin
β-actin
PDI
β-actin
95
43
72
43
95
43
55
43
C
DMSO
OLN
Relative protein level
1.5
1.0
0.5
0.0
*
n.s.
n.s.
n.s.
GRP94
HSP70
Calnexin
PDI

Figure S5

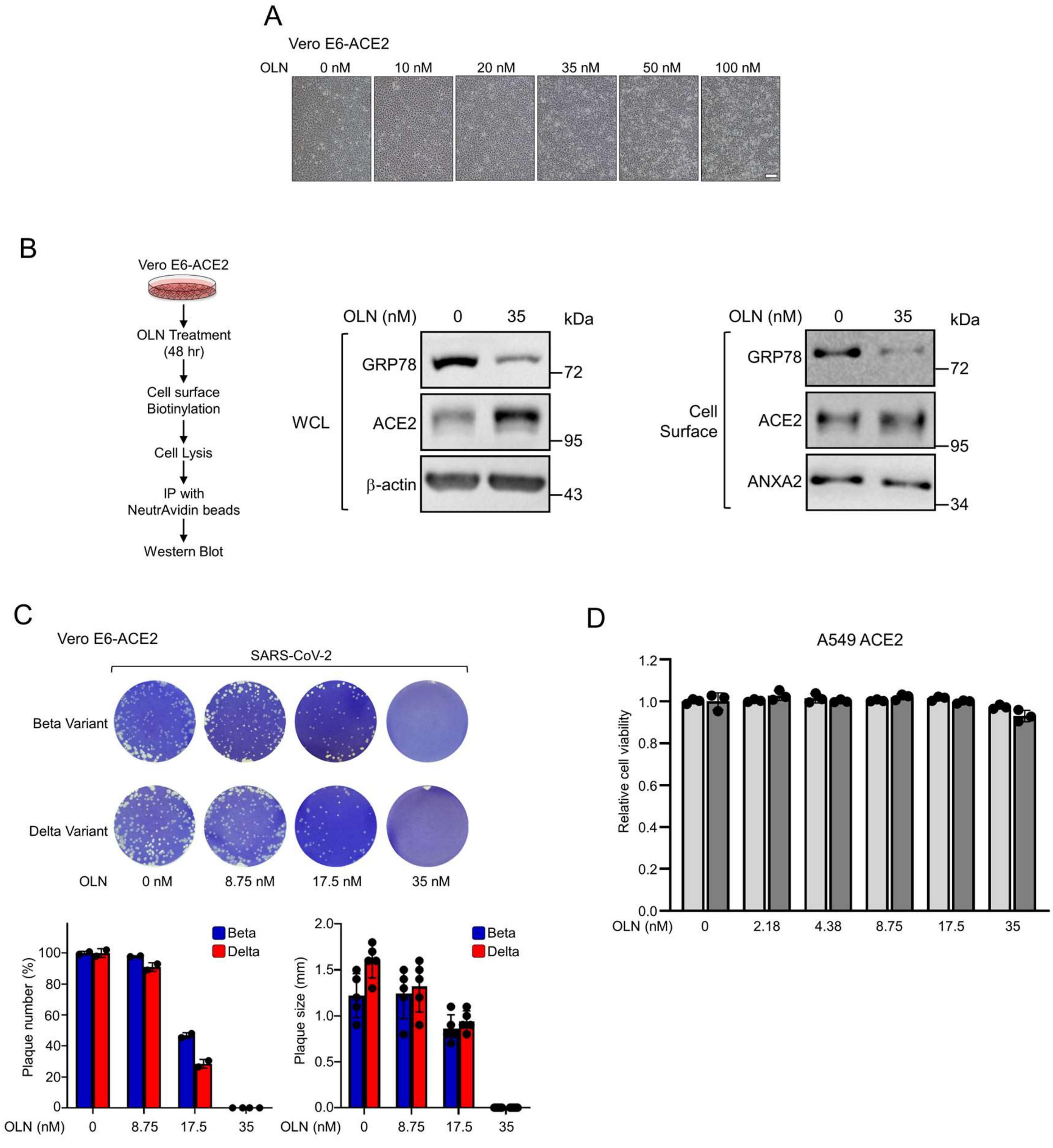
A
Vero E6-ACE2
OLN
0 nM
10 nM
20 nM
35 nM
50 nM
100 nM
B
Vero E6-ACE2
OLN Treatment (48 hr)
Cell surface Biotinylation
Cell Lysis
IP with NeutrAvidin beads
Western Blot
OLN (nM)
0
35
kDa
WCL
GRP78
ACE2
β-actin
72
95
43
Cell Surface
GRP78
ACE2
ANXA2
72
95
34
C
Vero E6-ACE2
SARS-CoV-2
Beta Variant
Delta Variant
OLN
0 nM
8.75 nM
17.5 nM
35 nM
Plaque number (%)
Beta
Delta
OLN (nM)
0
8.75
17.5
35
Plaque size (mm)
D
A549 ACE2
Relative cell viability
OLN (nM)
0
2.18
4.38
8.75
17.5
35

Figure S6

```
Wuhan           DYNYKLPDDFTGCVIAWNSNNLDSKVGGNYNYLYRLFRKSNLKPFERDISTEIYQAGSTP    479
Delta           DYNYKLPDDFTGCVIAWNSNNLDSKVGGNYNYRYRLFRKSNLKPFERDISTEIYQAGSKP    477
HK.3            DYNYKLPDDFTGCVIAWNSNKLDSKPSGNYNYLYRFLRKSKLKPFERDISTEIYQAGNKP    475
XBB.1.5.66      DYNYKLPDDFTGCVIAWNSNKLDSKPSGNYNYLYRLFRKSKLKPFERDISTEIYQAGNKP    476
JN.1.11.1       DYNYKLPDDFTGCVIAWNSNKLDSKHSGNYDYWYRSLRKSKLKPFERDISTEIYOAGNKP    475
                ********************:**** .***:* ** :***:****************..*

Wuhan           CNGVEGFNCYFPLQSYGFQPTNGVGYQPYRVVVLSFELLHAPATVCGPKKSTNLVKNKCV    539
Delta           CNGVEGFNCYFPLQSYGFQPTNGVGYQPYRVVVLSFELLHAPATVCGPKKSTNLVKNKCV    537
HK.3            CNGVAGPNCYSPLQSYGFRPTYGVGHQPYRVVVLSFELLHAPATVCGPKKSTNLVKNKCV    535
XBB.1.5.66      CNGVAGPNCYSPLQSYGFRPTYGVGHQPYRVLVLSFELLHAPATVCGPKKSTNLVKNKCV    536
JN.1.11.1       CKG-KGPNCYFPLQSYGFRPTYGVGHQPYRVVVLSFELLHAPATVCGPKKSTNLVKNKCV    534
                *:*  * *** *******:** ***:*****:****************************
```

Figure S7
