# Supplementary material for: Targeting stress induction of GRP78 by cardiac glycoside oleandrin dually suppresses cancer and COVID-19
Source: Cell Biosci. 2024 Sep 6;14:115. doi: 10.1186/s13578-024-01297-3 (PMC11378597; doi:10.1186/s13578-024-01297-3)
Supplement: Supplementary file 2 — Additional file 2: Table S1. GRP78 overexpression rescues virus release of SARS-CoV-2 under oleandrin treatment. Vero E6-ACE2 cells were transfected with pcDNA3 empty vector or FLAG-GRP78 for 48 hr. The cells were then infected with SARS-CoV-2 for 45 min followed by OLN treatments for 3 days. The titers of virus released into the media were quantified by plaque assay. [file 13578_2024_1297_MOESM2_ESM.pdf]

Table S1. Effect of GRP78 on the anti-SARS-CoV-2 activity of oleandrin.

Oleandrin concentration	35nM	17.5nM	8.75nM	4.38nM	0nM
Vector*	10±2	26±5.29	$5.67x10^2$±4.62x10	$1.6x10^5$±$8.08x10^4$	$1.67x10^6$±$6.11x10^5$
GRP78*	31.33±3.06	85.33±11.37	$2.22x10^3$±$1.14x10^2$	$2.35x10^5$±$2.35x10^4$	$1.66x10^6$±$7.21x10^5$
Ratio (GRP78/Vector)	3.13	3.28	3.93	1.46	0.99

* Values are pfu/ml, samples are titrated in triplicate

Table S1
